# Probabilistic modelling of prospective environmental concentrations of gold nanoparticles from medical applications as a basis for risk assessment

**DOI:** 10.1186/s12951-015-0150-0

**Published:** 2015-12-22

**Authors:** Indrani Mahapatra, Tian Yin Sun, Julian R. A. Clark, Peter J. Dobson, Konrad Hungerbuehler, Richard Owen, Bernd Nowack, Jamie Lead

**Affiliations:** School of Geography, Earth and Environmental Sciences, University of Birmingham, Edgbaston, Birmingham, B15 2TT UK; Empa–Swiss Federal Laboratories for Materials Science and Technology, Technology and Society Laboratory, Lerchenfeldstrasse 5, 9014 St. Gallen, Switzerland; Safety and Environmental Technology Group, Institute for Chemical and Bioengineering, ETH-Hoenggerberg, 8093 Zurich, Switzerland; The Queen’s College, Oxford, OX1 4AW UK; Warwick Manufacturing Group, University of Warwick, Coventry, CV4 7AL UK; Business School, University of Exeter, Exeter, EX4 4PU UK; Department of Environmental Health Sciences, Center for Environmental Nanoscience and Risk, Arnold School of Public Health, University of South Carolina, Columbia, SC 29208 USA

**Keywords:** Gold nanoparticles, Nanomedicine, Probabilistic modelling, Species sensitivity distribution, PEC, PNEC

## Abstract

**Background:**

The use of gold nanoparticles (Au-NP) based medical applications is rising due to their unique physical and chemical properties. Diagnostic devices based on Au-NP are already available in the market or are in clinical trials and Au-NP based therapeutics and theranostics (combined diagnostic and treatment modality) are in the research and development phase. Currently, no information on Au-NP consumption, material flows to and concentrations in the environment are available. Therefore, we estimated prospective maximal consumption of Au-NP from medical applications in the UK and US. We then modelled the Au-NP flows post-use and predicted their environmental concentrations. Furthermore, we assessed the environment risks of Au-NP by comparing the predicted environmental concentrations (PECs) with ecological threshold (PNEC) values.

**Results:**

The mean annual estimated consumption of Au-NP from medical applications is 540 kg for the UK and 2700 kg for the US. Among the modelled concentrations of Au-NP in environmental compartments, the mean annual PEC of Au-NP in sludge for both the UK and US was estimated at 124 and 145 μg kg^−1^, respectively. The mean PEC in surface water was estimated at 468 and 4.7 pg L^−1^, respectively for the UK and US. The NOEC value for the water compartment ranged from 0.12 up to 26,800 μg L^−1^, with most values in the range of 1000 μg L^−1^.

**Conclusion:**

The results using the current set of data indicate that the environmental risk from Au-NP used in nanomedicine in surface waters and from agricultural use of biosolids is minimal in the near future, especially because we have used a worst-case use assessment. More Au-NP toxicity studies are needed for the soil compartment.

**Electronic supplementary material:**

The online version of this article (doi:10.1186/s12951-015-0150-0) contains supplementary material, which is available to authorized users.

## Background

There has been an increased focus on developing gold nanoparticles (Au-NP) based applications in fields ranging from electronics to medicine. Between 2000 and 2013, gold nanotechnology related patents increased exponentially, with about 1600 patents published in 2013 [1]. The number of publications related to Au-NP in the health sector in Thomson Reuters’ Web of Science data base also show an exponential increase from 54 to 9083 publications between 2004 and 2014, of which 2150 articles were published in 2014 alone (search conducted on 28 Dec 2014) [2]. The unique chemical and physical properties of Au-NP [3–5] make them excellent candidates for exploitation in the medical field to help in disease diagnosis and treatment. Furthermore, their ease of synthesis in a variety of sizes and shapes and their amenability towards surface functionalization creates the possibility for multi-functionality including imaging and targeted drug delivery [6–10].

Drug delivery applications based on Au-NP are forecast to have a 21 % share of the USD 136 billion total market of nano-drug delivery applications by 2021 [11]. The enormous range of potential applications of Au-NP and their increased future use could result in greater risk of environmental release and exposure at low concentrations, as is the case with many pharmaceutical products [12–15]. Proliferation and increased application of single use and disposable cheap medical diagnostic devices [16] could add to this environmental burden.

Uptake, biodistribution, accumulation and biomagnification of Au-NP by environmental organisms have been studied by many investigators [17–19], and it has also been shown that Au-NP can be toxic to animals and plants [20–23] thus indicating that these supposedly biocompatible materials could present a significant hazard to plants and wildlife. Au-NP have been shown to have different modes of action for creating toxic effects dependent on their properties and the organism studied [24, 25] and show promise as an antibacterial agent [26].

In terms of environmental risks, studies on potential flows and concentrations of Au-NP in anthropogenic and ecological systems are non-existent. Overall there is limited environmental hazard data and no exposure data, making risk assessment highly problematic. Since there is potential for an exponential increase in use of Au-NP, it is timely to model their environmental flows and concentrations to help frame the risk analysis [27, 28], as has been done also for other nanomaterials [29–32].

In this study we have estimated the environmental concentrations of Au-NP for the United Kingdom (UK) and for the United States of America (US) from selected medical applications that are currently on the market or have potential to be introduced in the near future by developing a conceptual environmental exposure model and by combining this with the hazard data. Since no measured environmental concentration data is available for Au-NP, we have used probabilistic material flow analysis [33] to track the flow and fate of Au-NP during use and disposal as a first step to establish the possible future baseline in a worst case Au-NP release scenario. This approach attempts to address the uncertainty and variability in the data by creating probability distributions for all input data as has been described before [33, 34] Where there is limited toxicity data and where experimental procedures and methodologies have variability, use of probabilistic/stochastic methods to establish and quantify environmental risks can help to increase the robustness of the risk quotients. Thus, probabilistic species sensitivity distribution (pSSD) for quantifying ecotoxicological risks and comparing the modeled PEC to the predicted no adverse effect concentration (PNEC) based on toxicity data for the corresponding environmental compartment, forms the basis of our approach to derive risk levels for the ecosystem [35].

## Results and discussion

### Estimation of nano gold consumption from prospective medical applications

Table 1 details the estimated quantity of Au-NP from nano-enabled medical applications. As the table depicts, very small amounts—in the range of milligram to less than a few kilograms—are estimated to originate from in vitro medical devices or devices used for detection of specific disease biomarkers. Larger quantities of Au-NP are estimated to be released from applications used for treating or managing a particular disease, for example, for the treatment of gum infections, cancer and diabetes. The amount of Au-NP per patient was estimated to range from 0.05 mg to 5000 mg for the whole treatment cycle, the higher values corresponding to the treatment modality of photothermal ablation of cancer using gold nanoshells. A study [36] conducted in Northwest England estimated the consumption of anticancer drugs from hospital records and showed total consumption of all the identified anticancer drugs to be around 350 kg. Thus, the annual Au-NP consumption amount in the range <1 kg to 250 kg could be reached in the near future for the UK for treatment of breast, lung, pancreatic and bowel cancer. This is because these diseases have high incidence rates, however, it needs to be kept in mind that we have used high release scenario of 100 % patient access and treatment by the same Au-NP based therapeutic for all patients.

**Table 1:** Prospective amount (per annum) of Gold nanoparticles in selected medical applications (high release scenario)

Application	Consumption		Waste compartment
	UK	US	
Lab based lateral flow assay to detect the presence of Methicillin Resistant and Methicillin Sensitive *Staphylococcus aureus* in blood	0.34	6	Hazardous Medical/Clinical/Infectious Waste (HMCIW)
In vitro lab based diagnostic test kit for detection and genotyping warfarin metabolism	0.36	3	HMCIW
In vitro lab based diagnostic test kit for detection of single nucleotide polymorphism to detect risk from venous thrombosis	1	3	HMCIW
OTC pregnancy and ovulation test kits to detect hormones in urine	3–100	20–460	Municipal solid waste
Lab based in vitro rapid test kits for qualitative detection of antibodies to HIV-1 and HIV-2 in human serum, plasma and blood	2–80	20–830	HMCIW
Home based in vitro HIV test kits	20	90	Municipal solid waste
Lab based in vitro tests for detection of CD4 cells and viral loads for HIV patients	60	540	HMCIW
Lab based diagnostic test kits for infectious diseases	70	350	HMCIW
Removal of *Staphylococcus aureus* from the nasal passage of patients to reduce risks of nosocomial infections	30–53,300	110–164,640	HMCIW
Treatment of periodontitis	270–106,560	940–365,160	Waste water
Sensors for diagnosing diseases from breath samples	0.01–1590	0.03–4620	HMCIW
Treatment for solid tumors (colorectal, pancreas, breast)	70 -(480) -1100	310-(2020)–4600	Waste water
Last line treatment for patients with solid tumors (colorectal, pancreatic and breast)	420	1500	Waste water
Treatment for patients diagnosed with head and neck and lung cancer	140,290–233,820	744,750–1,241,260	Waste water
Last line treatment for patients with head and neck and lung cancer	104,710–174,520	468,250–780,410	Waste water
Transbuccal insulin delivery platforms	128,250	841,620	Waste water

The Table presents total gold nanoparticles consumption per annum for the UK and US using a worst case scenario. Data rounded off to 2 significant digits for values below 1 or data rounded off to the nearest integer or ten. Unit: gram. Refer to Additional file 1: Section S2 Estimation of annual Au-NP consumption for details related to assumptions and references

The Au-NP consumption data could be estimated due to the strict regulatory governance framework associated with approval of pharmaceutical products for human use and also because of the availability of disease incidence and prevalence data for widespread diseases, such as cancer, diabetes. In contrast, estimating Au-NP quantities from in vitro diagnostic devices was challenging due to the dependence on the patenting literature, wherein specific details are obscured and also because of the less stringent regulatory pathway for in vitro medical devices. Hence, the estimated data relied on vast number of assumptions and data was extrapolated from various literature sources.

### Mass flows of Au-NP

The annual mean prospective Au-NP use estimates for the UK and US are 540 kg and 2700 kg respectively. The yearly disease incidence rates of HIV/AIDS and cancer were found to be relatively stable over the last few years [37–41], so the data estimated in this study (which uses incidence and prevalence data compiled in the recent national disease registries and are for the years between 2007 and 2014) can be assumed to remain constant for the next 5 years. By combining the estimated maximal possible consumption of Au-NP with the technical and environmental transfer coefficients, we were able to obtain Au-NP flows from the end user to technical compartments and then further to receiving environmental compartments. Currently this represents an unrealistically high use of Au-NP and therefore our PEC values also represent highest possible concentrations. If Au-NP based applications for the healthcare sector are realised over the coming years, it may result in very high market penetration. For example, seven in vitro diagnostics, based on Au-NP for determining pregnancy and ovulation, were approved by the USFDA between 2009 and 2012. In our current assessment, only two uses dominate the overall Au-NP flows, a cancer treatment and an insulin delivery platform. The overall flows are therefore to a large extent following the flows of Au-NP used in these two applications, with all other uses having only a minor influence on the mean values but influencing the overall distribution and therefore the extreme values.

**Fig. 1:**
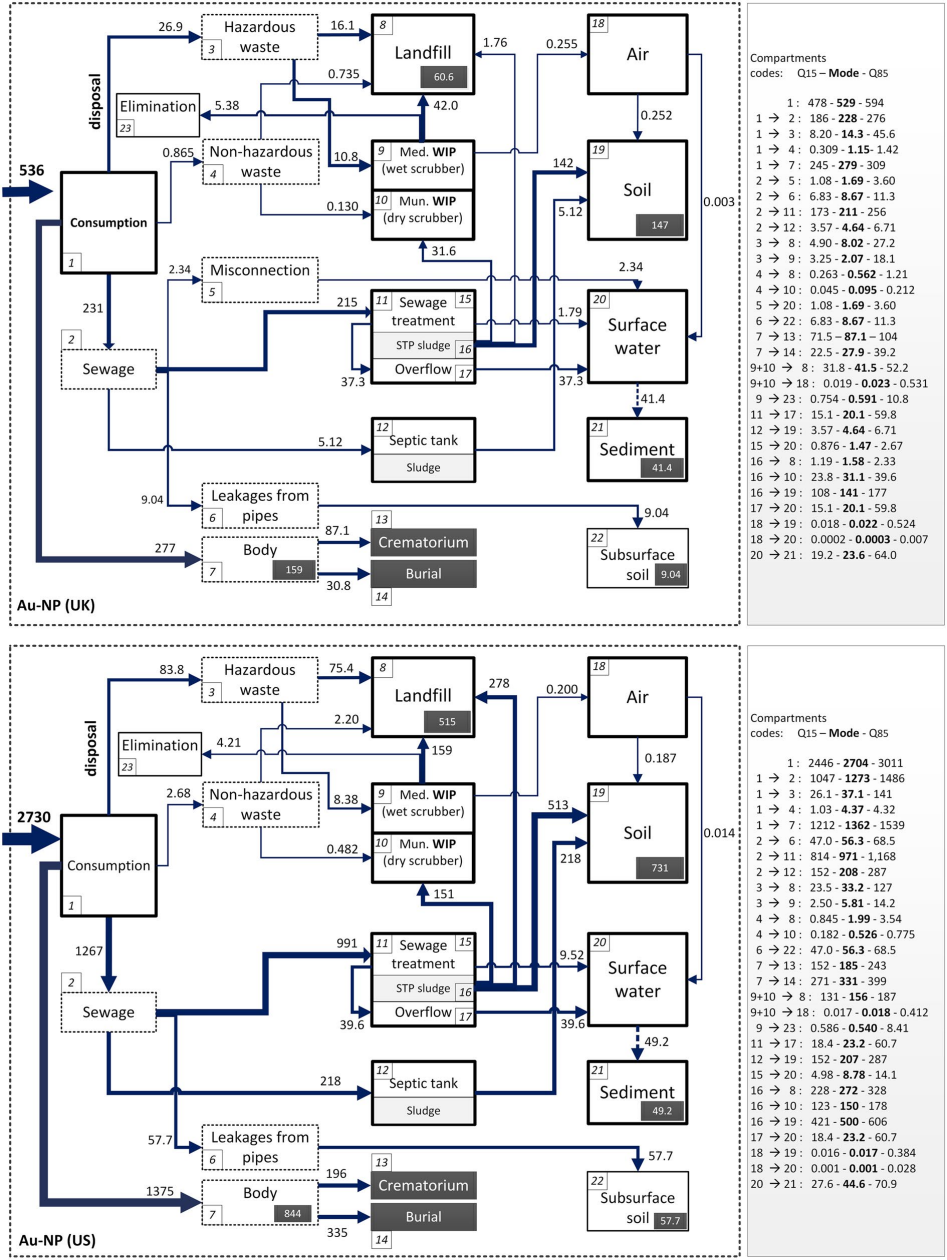
Modelled annual prospective mass flows (in kg) of Au-NP in the UK and US. Technical and environmental compartments are expressed as boxes and flows are expressed as arrows. The flow volumes used are mean values from the probability distribution of each flow. Each *box* (compartment) is given a code. Mean values, mode, quantile 15 (Q_15_) and Quantile 85(Q_85_) values are also given. These are indicated with compartment codes on the right side of the flowchart. The flow volumes are visualised by the thickness of the *arrows*. The compartments which we assumed to be the final sink are indicated by a *black square box* (body of living patients, crematorium, burial, landfill, soil, sediments and subsurface soils). Complete Au-NP suspension in surface water and complete Au-NP sedimentation from surface water to sediment are assumed in the calculation of mass flow (indicated by *dashed arrow*) and concentrations

Figure 1 shows that the most prominent Au-NP flows arise from consumption, leading to accumulation in the human body for both the UK and US. Based on pre-clinical data, we assumed 35 % [42] and 85 % [43] accumulation of Au-NP in the body for the two cancer therapeutics used as model input data. For other Au-NP based applications we assumed 100 % excretion [44, 45]. Of the total yearly consumption of Au-NP, around 160 and 850 kg of Au-NP respectively for the UK and the US would remain in the body of treated patients.

The second largest flow of Au-NP for both the UK and US is via sewage to sewage treatment plants (STPs). About 230 and 1300 kg of Au-NP from the total consumption for the UK and US, respectively, end up in sewage. In the UK, small amounts of Au-NP are directly transported to surface water due to misconnections and overflows. No data about misconnection for the US could be found, hence we have not modelled this value, but it is a potentially important source of uncertainty. In addition to misconnections, leakages from sewer pipes result in Au-NP mass transfer to subsurface soils. Au-NP reaching the STP might additionally not flow into the STP due to overflow discharges during rainy seasons. Compared to the US, overflows for the UK are more significant; direct discharge to surface waters accounts for nearly one-fifth of the total Au-NP initially reaching STPs; whereas for the US only 0.04 % of the total Au-NP by-passes the STP and reaches the surface waters.

Significant removal of Au-NP into the sludge, for both regions, results in significant quantities of Au-NP entering STPs, ending up in biosolids, which is partially further distributed onto agricultural soils as a fertilizer. Total Au-NP inputs in soil were modeled to be around 150 and 730 kg/year for the UK and US respectively. For the UK, around 32 kg of Au-NP present in the sludge reach the municipal waste incinerators (MWIs)) and a negligible quantity pass to the landfill i.e. the majority is applied as sludge to land. For the US, of the 990 kg of Au-NP present in sludge from centralized treatment works, around 280 and 150 kg were estimated to reach the landfill and MWIs compartments respectively. Au-NP from decentralized systems such as septic tanks, cesspools, etc. can be released to land and/or surface water, or underground water, based on the implementation status of relevant regulations. We assumed all Au-NP passing through the decentralized systems end up in sludge treated soils.

The third major flow of Au-NP is to the hazardous waste compartment for both regions. For the UK, 60 % of the 27 kg of hazardous waste was estimated to reach landfill, with the remainder in hazardous medical/clinical/infectious waste (HMCIW) incinerator, whereas for the US, 90 % of the 84 kg of Au-NP in the hazardous waste end up in landfills. These values indicate that clinical waste treatment via incineration is not a prevalent practice for both regions, and hence there is a possibility of Au-NP becoming accumulated in landfills in the future. However, these values need to be treated with caution because of the scarcity of national scale data with regard to waste management from healthcare facilities. Comprehensive and updated reports for medical waste for the US were not available and we depended on extrapolations from data reported in non-peer reviewed literature sources (details in Additional file 1: Table AF.T3.2). For the UK, only one peer reviewed paper [46] containing data for the year 2007 was available. Furthermore, the difference in the healthcare and biological waste (H&B) generation data in the Eurostat database, updated on Dec 6, 2013 [47] and DEFRA [48] report for the years 2004, 2006, 2008 indicate the need for coherent definitions and reporting. H&B generation data in the Eurostat database for the year 2010 was approximately 3 times more than the waste generated in 2008. Since there was no publication from DEFRA for the year 2010, the data reported in the Eurostat database could not be verified/triangulated and the reason for the increase was undecipherable. This indicates the poor state of environmental reporting, monitoring and updating between national scale and regional scale databases and between organizations in the EU.

### Au-NP concentrations in technical and environmental compartments

Table 2 shows the predicted Au-NP concentrations in STP effluent, surface water, STP sludge, and yearly concentration in sediments and biosolid treated soils for the UK and US. The values presented are mean values, mode values (the most probable values) and their 15th and 85th percentiles (Q_15_ and Q_85_) from each distribution. When comparing the two regions, predicted Au-NP concentrations were higher in the UK in nearly all the compartments when compared to those in the US, except for STP sludge which shows similar mean concentrations. The predicted environment concentration (PEC) in surface water in the US is the lowest among all the modeled technical and environmental compartments for UK and US.

**Table 2:** Predicted Au-NP concentrations in technical and environmental compartments

		UK				US				Units
		Mean	Mode	Q_15_	Q_85_	Mean	Mode	Q_15_	Q_85_	
STP Effluent		440	360	220	670	140	130	71	200	pg/L
Surface water		470	270	210	730	4.7	4.0	2.7	6.8	pg/L
STP sludge		120	130	94	150	150	150	120	170	μg/kg
*Sludge treated soil*		*300*	*300*	*230*	*370*	*150*	*150*	*120*	*170*	*ng/kg*· *years*
*Sediment*		*290*	*170*	*130*	*450*	*5.0*	*4.5*	*3.0*	*8.0*	*ng/kg·years*
Hazardous waste		77	78	23	130	65	69	20	110	μg/kg
Medical WIP										
Fly ash		270	30	36	530	260	32	36	530	μg/kg
Bottom ash		200	25	27	410	200	26	27	400	μg/kg
Municipal WIP										
Fly ash		72	70	53	92	39	38	31	47	μg/kg
Bottom ash		55	52	39	71	30	27	22	37	μg/kg

The mean, mode (most probable values), quantile 15 (Q_15_) and quantile 85 (Q_85_) for the predicted concentrations in the technical environmental compartments are provided on the table. Values in italics designate yearly increases in concentrations. Au-NP concentrations in surface water and sediments represent no and complete sedimentation respectively. The results are expressed up to two significant digits

In the UK, the predicted Au-NP concentration in surface water is higher than in sewage effluent. This is due to the fact that a significant amount of Au-NP is estimated to be released directly to surface waters via overflows. In contrast, the lower Au-NP concentration in STP effluent and the lower PEC in surface water for the US can be explained by the much larger STP effluent volume produced per capita. According to USEPA, 625 liters of STP effluent is produced per capita per day [49] whereas for the UK, it is 150-180 liters per capita per day [50, 51] (see tables in Additional file 1). The mean modeled Au-NP concentration in surface waters for both regions is in the range of 5–470 pg L^−1^ which is similar to the background gold concentration reported in freshwaters (reviewed by McHugh [52]). PECs in surface water of Germany for iron oxide nanoparticles based MRI contrast agents were estimated to be 400 and 3140 pg L^−1^ for the year 2015 for two different scenarios used by author [53]. Measured environmental concentrations in surface waters of various anticancer drugs in use are in the range 500 to 41000 pg L^−1^ [36], indicating that the results of our model are at a similar level.

Predicted mean concentrations of Au-NP in STP sludge are 124 and 145 μg kg^−1^ for the UK and US, respectively. The PEC in sludge is considerably less than the measured total gold concentration of 790 μg kg^−1^ reported in a Swedish study [54]. The second highest concentration of Au-NP is in biosolid treated soils, although yearly concentrations are only in ng kg^−1^ levels. However, continuous application of biosolids on agricultural land might lead to Au-NP accumulation in soil over years. The lower predicted concentration of Au-NP in US agricultural soils is because of the larger area of the country and hence larger mass of biosolid treated agricultural soils in comparison to the UK.

The Au-NP concentrations for water and sediment concentrations are for worst-case scenarios, i.e., we did not model any fate in the environment but assumed that for the water compartment no sedimentation and for the sediment compartment complete sedimentation. Only a full environmental fate modelling including a mechanistic modelling of heteroagglomeration, sedimentation and transport will enable to predict the actual concentrations but these models [55–57] will rely heavily on input data to the environmental compartments that are provided by the material flow modelling carried out in this study. The environmental concentrations calculated in this work are valid for a regional assessment and are based on well-mixed compartments and follow as such the ECHA guidance [58]. A next step in the exposure assessment would be to regionalize the emissions which also allow to identify hotspots [59, 60].

### Risk assessment with probabilistic species sensitivity distribution (pSSD)

Aquatic species show a wide range of responses to Au-NP, with no observed effect concentrations (NOECs) ranging from 0.12 μg L^−1^ up to 26,800 μg L^−1^; a spread of five orders of magnitude, although most values are in the 1000 µg L^−1^range. The most sensitive species was the single cell green algae, *Chlamydomonas reinhardtii*, (an acute toxicity study done using 2 nm Au-NP capped with D-manno-pyranoside terminated PAMAM (polyamidoamine) G0 generation dendrimer) [23]. PAMAM dendrimers of different cores and generations (G2 to G6) have been shown to exert toxic affects in fish, freshwater crustaceans and algae with L(E)C_50_ values in the range 0.13–194 μM (reviewed in [61]).

**Fig. 2:**
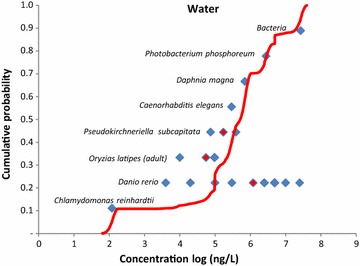
Probabilistic species sensitivity distribution (pSSD) for Au-NP for the water compartment. Probabilistic species sensitivity distribution (pSSD) for Au-NP in fresh water (*red line*) compared with the raw sensitivity data used (*blue diamond*). The red diamonds are the geometric means of the raw sensitivity data if there are more than one data available. The number of blue diamonds for each species corresponds to the number of raw sensitivity data available and used. The raw sensitivity data indicate the no observed effect concentrations (NOEC)

Figure 2 shows the cumulative probabilistic species sensitivity distribution (pSSD) for Au-NP in water. The results lacked sufficient resolution to decipher which taxa are most affected, and what particle properties are related to toxicity, though it seems fish (*Danio rerio*) were the least sensitive species when exposed to Au-NP in an aquatic environment. Publications with properly designed experiments [62, 63] or environmentally relevant exposure concentrations for studying toxic effects of Au-NP on environmental organisms are sparse. Barring a few, the studies selected do not report the L(E)C_x_ (lethal/toxic effect shown by x % of the organisms at a particular concentration) value, or the statistical method used to arrive at the reported data, do not mention acceptable control performance, and lack characterization of the NPs throughout the exposure duration. These results indicate the high variability of input model data, reflecting the varied toxic potential of Au-NP of different sizes and coating to different species. Therefore, reliable toxicity studies with specific Au-NP used for medical applications are needed for improved environmental risk assessment to influence policy makers for aiding regulatory decision making and responsible innovation [64]. It is also necessary to study the environmental stability and fate of the coatings of the Au-NP once released to wastewater or the environment.

By using probability distributions in place of single values we attempted to address the variability and the uncertainty which is inherent in toxicity studies. The hazard assessment we performed is for a “generic” Au-NP, considering all different sizes and coatings, representing the full width of currently used Au-NP in toxicity studies. This enables us to compare in a next step this “generic Au-NP SSD” with the modelling of the flows and concentrations which is also for a “generic Au-NP” because data on specific forms of Au-NP is not available.

**Fig. 3:**
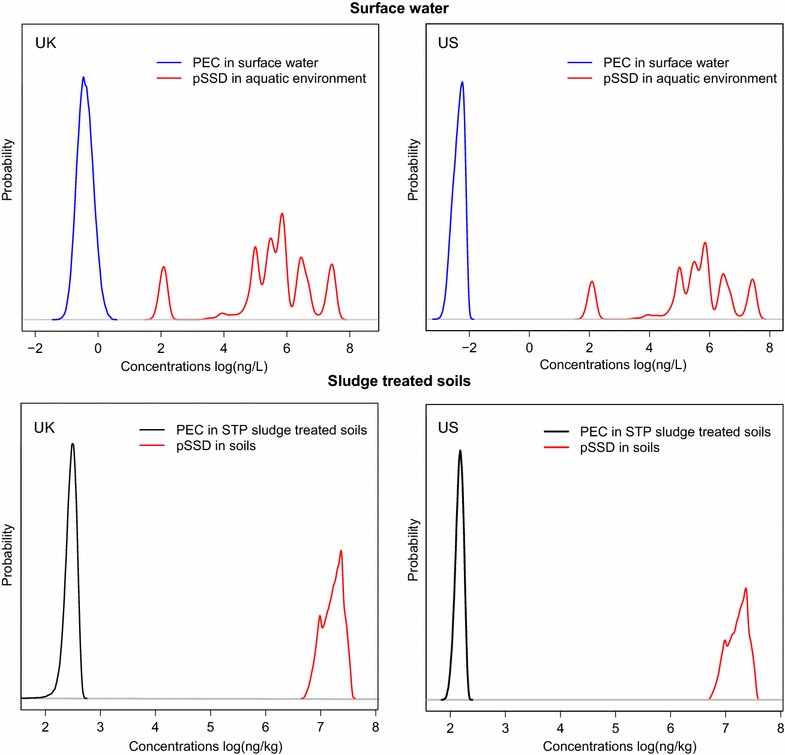
Predicted Environmental Concentration (PEC) and Predicted No-Effect Concentration (PNEC) distribution for surface water and sludge treated soils compartment. The PEC and pSSD distribution is in *blue* (water compartment) or* black *(soil compartment) and *red colour* respectively. Probabilistic species sensitivity distribution (pSSD) which reflects the no observed effect concentration data compared to the probability distributions of predicted environmental concentrations (PEC) of Au-NP in surface water and sludge treated soils in the UK and the US. Environmental risk could occur where the PEC overlaps the pSSD (not the case for Au-NP)

Figure 3 shows the probability distributions of the PECs and the pSSDs for Au-NP in the aquatic and terrestrial environment for both the UK and US. The PEC and pSSD for surface water and soils are compared and risks may arise where the PEC and pSSD overlap. It is clear that there is no overlap between the PEC and pSSD in both environmental compartments considered for the UK and US. The narrowness of the PEC probability density curves is due to the fact that few of the Au-NP application categories dominate the total consumption resulting in a narrow distribution of the total input into the system.

## Conclusion

Many human pharmaceuticals occur in the aquatic environment in ng L^−1^ concentrations [14, 65, 66] and studies have shown accumulation of these chemicals in aquatic organisms [15, 67, 68] and their adverse effects [13, 69]. The very defining property of nanoparticles—size and surface area—coupled with their ability to interact at subcellular levels to generate subtle biochemical changes [70], their novel properties and gaps in knowledge regarding relationship between chronic and acute toxicity, calls for the inclusion of sub-lethal toxicity endpoints for regulatory decision making. In one scenario we also included selected sub-lethal endpoints in the pSSD (results are provided in the Additional file 1 section S3: Alternate Scenarios) but the risk assessment does not significantly change.

Because nanomaterials have been found to undergo transformation both inside human body as well as the environment [71], their fate can change accordingly in real world situations. However, for Au-NP chemical degradation is rather unlikely due to the inert nature of gold but transformations of surface coatings will strongly affect environmental fate. This will be important when the results from our material flow modelling are used in environmental fate models which include a specific description of fate processes [55–57].

In an ideal situation environmental risk assessment should be based on a full characterization of the material and its transformation products; in the case of nanomaterials such complete risk assessments are not yet available [27]. The complex challenge can currently be addressed in a number of ways, for example by using expert judgment and multi-criteria decision analysis [72, 73] and species sensitivity distributions [74] for different types of a nanomaterial. The probabilistic risk assessment using both probabilistic species sensitivity distributions and probabilistic mass flow models enables to consider the complete current knowledge in a systematic and comprehensive way and has been applied to other ENM before [75, 76]. Both exposure and hazard data are limited and the model provides a way to deal with this uncertainty. Extensive literature search combined with communications with experts in the field has helped us to arrive at plausible estimates. The results from the model can be used to provide a baseline for realistic and environmentally relevant exposure/toxicology studies and can help in iterative problem formulation and solution, as more concrete data becomes available. The modelling performed here suggest that freshwater (and hence sediments) and biosolids treated soils would likely receive highest loads of Au-NP for the UK. Risk from Au-NP to aquatic organisms and soil organisms seems to be unlikely in the near future at a regional scale, although variations will exist temporally and spatially and can also be influenced by the presence of natural Au-NP [77].The study models for high loading of Au-NP and depends on worst case assumptions with regard to environmental transformation and fate, hence real concentrations in the environment are likely to be much lower. Developing environmental fate models and models addressing temporal and spatial issues can be a possible next step to arrive at more robust estimates of Au-NP concentration in the environment. Hazard assessment data for soil organisms is severely limited and so uncertainty is particularly high indicating that more Au-NP toxicity research is needed for soil organisms. Empirical fate and transformation data of Au-NP for incinerators as well as freshwater systems is non-existent and research is needed for Au-NP transformation in STPs with different treatment processes using Au-NP with surface coatings used in medical applications.

## Methodology

### General model layout

We have used the geographical regions of the UK and US (excluding dependent areas) as the units of analysis for our study. Similar to the approach proposed by the Guidelines for environmental risk assessment (ERA) of human pharmaceuticals [78, 79], (hereinafter referred to as ‘Guidelines’) where the consumption data of a drug per year is the key input factor, the model input in this study is based on population based estimates of use and consumption of the selected medical applications in a given year and disregards the manufacturing and processing facilities as a potential source. The model is a step-wise process where the selected application’s post usage life cycle has been mapped through the technical compartments of STPs, waste incineration plants (WIPs), landfills and the environmental compartments of soil, water and sediments. In addition to Au-NP based therapeutic agents which are in early stages of clinical trials, we have estimated Au-NP concentrations in medical devices approved by regulatory agencies or in late stages of product development. A deviation from the Guidelines is the use of excretion rates from pre-clinical studies as opposed to assuming 100 % excretion. We have considered possible variable retention of Au-NP in STPs. PECs in various compartments and risk assessment results considering 100 % excretion are provided in the Additional file 1 under section 3: Alternate Scenarios. The data and values used to arrive at gold amounts per use are based on broad estimates derived from the available literature and the patient population and hence the study is a bottom up, high release scenario study. We have assumed Au-NP to be spherical in shape and have used mass concentrations to estimate consumption amounts.

Transfer coefficients (TC) have been used to model the behaviour of Au-NP in various environmental and technical compartments included within the model (see Fig. 1 for details). The data used in the model have high uncertainty, compounded by large variability and hence we built probability distributions for the majority of input data. Estimated consumption values of products which have the same life-cycle pathway have been summed by adding their individual probability distributions. Additional file 1: Table AF.T1 illustrates the probability distributions for all data used in the study.

To estimate the volumes of the environmental compartments, we have used ECHA’s guidance on environmental exposure estimation for chemicals for a regional scale model [58]. The mass and volumes along with the assumptions of the transition and final environmental compartments are detailed in Additional file 1: Tables AF.T3.1, AF.T3.2 and AF.T3.3. Seawater is not included in our model. The assumptions of a well-mixed, homogenous and stationery system have been applied in this study which is a standard approach to arrive at crude estimates of environmental concentrations at a regional level [29]. The model tracks the Au-NP mass and not the total gold mass. Loss of the nano-property (e.g. by vaporization) therefore constitutes an elimination flow.

### Methodological approach for input data

An extensive literature search was carried out to identify relevant peer reviewed scientific publications of Au-NP or gold colloids in the medical field, administration doses, distribution, excretion, environmental fate and behaviour and environmental toxicity. Our aim was to identify Au-NP enabled medical applications which are approved, in clinical trials or show promise of translation from pre-clinical models. Reports published by UK and US Government Department and Agencies have been relied upon for estimating population, environment and technical compartment data. The transfer coefficients have been estimated by reviewing literature and/or soliciting expert viewpoints. Triangulations between various publications were performed and the approach of the best available data was adopted to arrive at the estimates used in this study. Details regarding consumption data and assumptions and references therein are included in the Additional file 1 section 2: Estimation of annual Au-NP consumption and Additional file 1: Table AT.T2.

### Transfer factors

Therapeutics based on Au-NP, after use, will end up either in solid waste, when the containers with the remnants of the therapeutic and associated procedural implements are disposed of as part of HMCIW and/or in the sewerage system when it is excreted from the body in urine or faeces. In vitro diagnostic devices used in hospitals and other healthcare settings will likely be part of HMCIW. Over-the-counter (OTC) single use medical devices are likely to end up in household waste. Therefore, wastewater (WW)/sewerage, HMCIW and household waste are defined as the key potential sources of entry of Au-NP from medical products to the environment.

#### Au-NP flow into sewage treatment plants and surface water

Not all houses are served by a centralised STP. The connection rates to STP are 96 % [80] and 74 % [81] for the UK and the US respectively. Untreated sewer overflows, misconnections whereby grey water from households is connected to the storm water drainage systems, and exfiltration from sewerage pipes can result in untreated WW reaching surface waters, groundwater and subsurface soil directly. Au-NP from WW can also enter the environment due to failure of decentralised STPs. Since the connection rate to STPs for the UK is 96 %, we have neglected the contribution of individual septic tanks, cesspools, etc. to the pollution load. However, for the US, nearly 25 % of the total population is served by decentralised systems and the USEPA suggests a failure rate of 6 % annually of these systems [82]. Therefore, for the US we have considered failures of decentralised systems as a source of Au-NP reaching the environment. Additionally, discharge of untreated WW due to the dilapidated state of sewerage infrastructure [83] and polluted outfalls from combined sewers during rains [81] can add to the pollution load of surface waters.

#### Behaviour of Au-NP in surface water

Data was non-existent with regard to Au-NP fate in surface waters and we have therefore modelled two extreme scenarios to represent worst case conditions for both compartments. We assumed that Au-NP entering the surface freshwater compartment were either 100 % deposited to the sediment to derive sediment concentrations, or remained 100 % in the water phase to derive freshwater concentrations.

#### Behaviour of Au-NP in Sewage Treatment Plant

Only one published study is available where an estimate of the removal efficiency of Au-NP in STPs has been provided [84]. This study found 99 % removal rate of polymer coated Au-NP of sizes 10 nm and 100 nm in activated sludge batch experiments irrespective of coating, sizes and treatment. We have therefore used a removal efficiency of 99 % for wastewater treatment. However, we acknowledge that removal efficiencies will differ based on the WW treatment systems used [85, 86].

#### Au-NP flow into waste compartment

Household waste is non-hazardous in nature and hence in addition to incineration, discarding to landfill is another preferred mode of treatment. OTC disposable in vitro diagnostic devices containing Au-NP will be part of the household and similar waste category as defined in the European Union Waste catalogue [87]. In the UK, the proportion of landfilled and incinerated waste for the category of household and similar waste is 85 and 15 % respectively for the year 2008 [47]. For the US, the proportion of household waste sent to landfill and incinerated is 82 and 18 % respectively of the total waste discarded after the recovered fraction [88].

Wastes from healthcare settings are both hazardous and non-hazardous in type. Hazardous waste from healthcare facilities are generally sent for high temperature treatments like incineration and pyrolysis, or alternatively non-burn low temperature treatments or chemical treatments to disinfect the infectious waste [46]. These alterative treatment technologies use wet or dry steam at temperatures lower than 200 °C and use chemical disinfection methods. We have assumed that Au-NP will not be transformed/destroyed when waste is treated via non-burn alternative treatment technologies and will eventually end up in landfill.

#### Behaviour of Au-NP during Waste Incineration

No information is available about the fate of Au-NP in incinerators. Depending on the type of waste, type of incinerator and operating temperatures, configuration of the air pollution control devices (APCDs), and the particle size, it is likely that Au-NP will partition into bottom ash, APCD residues and stack emissions from APCDs.

Emissions from incinerators are under strict regulatory control; therefore it has been assumed that all municipal waste and HMCIW incinerators will have associated APCDs. Both the UK and US use dry or semi–dry scrubbing systems with fabric filters or electrostatic precipitators (ESPs) as the main types of APCDs in the municipal waste incinerators [89, 90].

The temperatures in HMCIW incinerators having secondary chambers can reach as high as 1100 °C, which is higher than the melting temperature of bulk gold. Melting temperature depression related to particle size, both for free Au-NP and substrate supported Au-NP, has been proven by many investigators [91–95]. Furthermore, the presence of chlorine generated from Polyvinyl chloride in the incinerator can increase metal volatility and release into gas phase [96]. The vapour pressure of gold at 1095 °C is about 1 × 10^−5^ torr (1.33 × 10^−3^ Pa) [97] and that means typically around one monolayer of gold will be vaporized in 0.1 s. Hence, Au-NP entering the HMCIW incinerators will either melt or vaporize. In both cases the nano-property of the gold is lost and the Au-NP is no longer distinguishable from the other gold forms. We have used both the case of 0 and 100 % elimination of the gold mass. In the case of 0 % elimination, we assume Au-NP to be distributed 81 % in the bottom ash and 19 % in the fly ash using the values found by Walser et al. [98] for removal of Ceria nanoparticles in municipal waste incinerators. Of the 19 % of Au-NP in the fly ash, we assume 50 % of the Au-NP pass through the wet scrubbers and the remaining 50 % through the fabric filter for both the UK and US. This assumption was extrapolated from the type of APCD installed in the HMCIW incinerators in the US [99] since no data was available with regard to APCDs for HMCIW incinerators in the UK.

The operating temperatures in municipal waste incinerators are around 850 °C, so we assume that 81 % of Au-NP mass will be removed in the bottom ash and 19 % in the fly ash [98] of which 99.99 % will be removed by the ESP and fabric filter as APCD residue. These residues are treated as hazardous waste and are finally disposed to secured landfills or abandoned underground mines [100]. Bottom ash from municipal waste combustors can be used in the construction sector [101]. However, due to non-uniformity in available data for the selected regions and to simplify the model, we have neglected bottom ash recycling rate and have presumed that 100 % of the bottom ash from both types of incinerators will be landfilled.

We have not included the leachate from landfill and subsequent contamination of the ground water compartment because studies on the fate of nanoparticles in landfills are not yet available. The technical compartment of cremation has been considered in the model boundary with the assumption that some percentage of Au-NP might remain in the human body post treatment when Au-NP has been administered as a last line treatment. The temperature in crematoria is not high enough to vaporize or melt Au-NP [102] and hence we assume that untransformed Au-NP will form part of the ash.

Therefore, human body, landfills, sediments, subsurface soils and burial grounds have been considered as the final sink of the product life cycle post usage.

### Ecological risk assessment

To derive species sensitivity distributions for environmental effects of Au-NP, an extensive search of the ecotoxicological literature was conducted. Fourteen relevant studies were found published between 2008 and Feb 2014. Twenty-six data points across five taxonomically different environmental organisms—bacteria, fish, algae, crustacean and ciliates—were included in the assessment. The endpoints used were mortality and malformations, growth inhibition and reproductive performance. These endpoints were selected to maximize utility of the data points from the available published literature and because these endpoints can impact species survival. We considered all endpoints reported in a study even if they used different particle size and coating with the aim to create a generic Au-NP species sensitivity distribution to compare with the PEC of Au-NP which considers the mass of Au-NP. If in a study only one concentration has been tested on an organism and it had shown no effect for the selected toxicity endpoint, we have used that concentration as no-observed-effect concentration (NOEC), acknowledging that this could in reality be higher. When a range of concentrations were tested [103, 104], the highest concentration at which no statistically significant adverse effect was observed was used as the highest-observed-no-effect-concentration (HONEC). The raw data were converted to species sensitive values below which long-term negative impacts on the species were considered to be excluded using two assessment factors (AF) based on the REACH guidelines [105]. The first AF was used to convert acute toxicity to chronic toxicity (AF time = 1, in the case of chronic and long-term test; AF time = 10, in the case of acute and short-term test). All but two data points represented acute or short-term exposures. The second AF was used to convert the various endpoints to NOEC values (AF no effect = 1 for NOEC, AF no-effect = 2, if L(E)C_10_ ≤ L(E)Cx < L (E)C _50_ and AF = 10, if L(E)_50_ ≤ L(E)Cx ≤ L(E)C _100_). In studies where effect concentrations were reported in terms of molar concentrations, we have converted the values to mass concentration (μg/L), because regulatory limits are expressed as such. The studies selected and the associated end points arranged species wise are detailed in Additional file 1: Tables AF.T4.1, AF.T4.2. Probabilistic species sensitivity distributions were constructed for soil and freshwater as explained in an earlier study [35].

## Additional file

10.1186/s12951-015-0150-0Supporting Information

## Abbreviations

AF: assessment factors
APCDs: air pollution control devices
DEFRA: Department for Environment, Food and Rural Affairs
ECHA: European Chemicals Agency
ERA: environmental risk assessment
ESPs: electrostatic precipitators
EU: European Union
Au-NP: gold nanoparticles
H&B: healthcare and biological
HIV/AIDS: human immunodeficiency virus/acquired immunodeficiency syndrome
HMCIW: hazardous medical/clinical/infectious waste
HONEC: highest-observed-no-effect-concentration
L(E)C_x_: lethal (adverse effect) concentration, when x % of the test organisms die or are adversely effected
LOEC: lowest observed effect concentration
MRI: magnetic resonance imaging
MWIs: municipal waste incinerators
NOEC: no-observed-effect concentration
OTC: over-the-counter
PAMAM: polyamidoamine
PEC: predicted environment concentration
PNEC: predicted no-effect concentration
pSSD: probabilistic species sensitivity distribution
REACH: Registration, Evaluation, Authorisation and restriction of Chemicals
SI: supporting information
TC: transfer coefficients
UK: United Kingdom
US: United States of America
USFDA: United States Food and Drug Administration
USEPA: United States Environment Protection Agency
WW: waste water
WIPs: waste incineration plants

## References

[1] World Gold Council. Number of published patents including the words ‘gold’ and ‘nanoparticles’. http://www.gold.org/advanced_by_gold/#!science#gold-applications-en. Accessed 4 Jan 2015.

[2] Title search = (health* or medic* or therap* or diseas* or cancer* or HIV or AID*) AND title search = (nano* or ultra small) AND (gold or Au) -Time period: 2004-2014 [database on the Internet]. Accessed: 28 December 2014.

[3] Eustis S, El-Sayed MA (2006). Why gold nanoparticles are more precious than pretty gold: noble metal surface plasmon resonance and its enhancement of the radiative and nonradiative properties of nanocrystals of different shapes. Chem Soc Rev.

[4] Masitas RA, Zamborini FP (2012). Oxidation of highly unstable <4 nm diameter gold nanoparticles 850 mV negative of the bulk oxidation potential. J Am Chem Soc.

[5] Trudel S (2011). Unexpected magnetism in gold nanostructures: making gold even more attractive. Gold Bull.

[6] Lukianova-Hleb EY, Ren X, Sawant RR, Wu X, Torchilin VP, Lapotko DO (2014). On-demand intracellular amplification of chemoradiation with cancer-specific plasmonic nanobubbles. Nat Med.

[7] Shilo M, Motiei M, Hana P, Popovtzer R (2014). Transport of nanoparticles through the blood–brain barrier for imaging and therapeutic applications. Nanoscale.

[8] Setua S, Ouberai M, Piccirillo SG, Watts C, Welland M (2014). Cisplatin-tethered gold nanospheres for multimodal chemo-radiotherapy of glioblastoma. Nanoscale.

[9] Kircher MF, de la Zerda A, Jokerst JV, Zavaleta CL, Kempen PJ, Mittra E et al. A brain tumor molecular imaging strategy using a new triple-modality MRI-photoacoustic-Raman nanoparticle. Nat Med. 2012;18(5):829–34. http://www.nature.com/nm/journal/v18/n5/abs/nm.2721.html#supplementary-information. 10.1038/nm.2721 PMC3422133 22504484

[10] Arnaiz B, Martinez-Avila O, Falcon-Perez JM, Penades S (2012). Cellular uptake of gold nanoparticles bearing HIV gp120 oligomannosides. Bioconj Chem.

[11] Cientifica Ltd. Market opportunities in nanotechnology drug delivery. online. London, UK 2012.

[12] Ramirez AJ, Brain RA, Usenko S, Mottaleb MA, O’Donnell JG, Stahl LL (2009). Occurrence of pharmaceuticals and personal care products in fish: results of a national pilot study in the United States. Environ Toxicol Chem.

[13] Jobling S, Owen R. Ethinyl oestradiol in the aquatic environment. Copenhagen, Denmark: European Environment Agency 2013. Report No.: No. 1, Vol. 13.

[14] Roberts PH, Thomas KV (2006). The occurrence of selected pharmaceuticals in wastewater effluent and surface waters of the lower Tyne catchment. Sci Total Environ.

[15] Miller TH, McEneff GL, Brown RJ, Owen SF, Bury NR, Barron LP (2015). Pharmaceuticals in the freshwater invertebrate, Gammarus pulex, determined using pulverised liquid extraction, solid phase extraction and liquid chromatography–tandem mass spectrometry. Sci Total Environ.

[16] Keel T (2013). Gold and diagnostics—some staggering numbers. Gold Bull.

[17] Ferry JL, Craig P, Hexel C, Sisco P, Frey R, Pennington PL et al. Transfer of gold nanoparticles from the water column to the estuarine food web. Nat Nano. 2009;4(7):441–4. http://www.nature.com/nnano/journal/v4/n7/suppinfo/nnano.2009.157_S1.html. 10.1038/nnano.2009.157 19581897

[18] Judy JD, Unrine JM, Bertsch PM (2011). Evidence for biomagnification of gold nanoparticles within a terrestrial food chain. Environ Sci Technol.

[19] Sabo-Attwood T, Unrine JM, Stone JW, Murphy CJ, Ghoshroy S, Blom D (2012). Uptake, distribution and toxicity of gold nanoparticles in tobacco (Nicotiana xanthi) seedlings. Nanotoxicology.

[20] Tsyusko OV, Unrine JM, Spurgeon D, Blalock E, Starnes D, Tseng M (2012). Toxicogenomic responses of the model organism caenorhabditis elegans to gold nanoparticles. Environ Sci Technol.

[21] Kim KT, Zaikova T, Hutchison JE, Tanguay RL (2013). Gold nanoparticles disrupt zebrafish eye development and pigmentation. Toxicol Sci Off J Soc Toxicol.

[22] Geffroy B, Ladhar C, Cambier S, Treguer-Delapierre M, Brethes D, Bourdineaud JP (2012). Impact of dietary gold nanoparticles in zebrafish at very low contamination pressure: the role of size, concentration and exposure time. Nanotoxicology.

[23] Perreault F, Bogdan N, Morin M, Claverie J, Popovic R (2012). Interaction of gold nanoglycodendrimers with algal cells (*Chlamydomonas reinhardtii*) and their effect on physiological processes. Nanotoxicology..

[24] Cui Y, Zhao Y, Tian Y, Zhang W, Lu X, Jiang X (2012). The molecular mechanism of action of bactericidal gold nanoparticles on *Escherichia coli*. Biomaterials.

[25] Coradeghini R, Gioria S, García CP, Nativo P, Franchini F, Gilliland D (2013). Size-dependent toxicity and cell interaction mechanisms of gold nanoparticles on mouse fibroblasts. Toxicol Lett.

[26] Zhao Y, Tian Y, Cui Y, Liu W, Ma W, Jiang X (2010). Small molecule-capped gold nanoparticles as potent antibacterial agents that target Gram-negative bacteria. J Am Chem Soc.

[27] Owen R, Handy RD (2007). Viewpoint: formulating the Problems for Environmental Risk Assessment of Nanomaterials. Environ Sci Technol.

[28] Pastoor TP, Bachman AN, Bell DR, Cohen SM, Dellarco M, Dewhurst IC (2014). A 21st century roadmap for human health risk assessment. Crit Rev Toxicol.

[29] Gottschalk F, Sonderer T, Scholz RW, Nowack B (2009). Modeled environmental concentrations of engineered nanomaterials (TiO2, ZnO, Ag, CNT, Fullerenes) for different regions. Environ Sci Technol.

[30] Sun TY, Gottschalk F, Hungerbuhler K, Nowack B (2014). Comprehensive probabilistic modelling of environmental emissions of engineered nanomaterials. Environ Pollut.

[31] Keller A, McFerran S, Lazareva A, Suh S (2013). Global life cycle releases of engineered nanomaterials. J Nanopart Res.

[32] Keller AA, Lazareva A (2014). Predicted releases of engineered nanomaterials: from global to regional to local. Environ Sci Technol Lett.

[33] Gottschalk F, Scholz RW, Nowack B (2010). Probabilistic material flow modeling for assessing the environmental exposure to compounds: methodology and an application to engineered nano-TiO2 particles. Environ Model Softw.

[34] Gottschalk F, Sonderer T, Scholz RW, Nowack B (2010). Possibilities and limitations of modeling environmental exposure to engineered nanomaterials by probabilistic material flow analysis. Environ Toxicol Chem.

[35] Gottschalk F, Nowack B (2013). A probabilistic method for species sensitivity distributions taking into account the inherent uncertainty and variability of effects to estimate environmental risk. Integr Environ Assess Manag.

[36] Booker V, Halsall C, Llewellyn N, Johnson A, Williams R (2014). Prioritising anticancer drugs for environmental monitoring and risk assessment purposes. Sci Total Environ.

[37] Prejean J, Song R, Hernandez A, Ziebell R, Green T, Walker F (2011). Estimated HIV incidence in the United States, 2006–2009. PLoS One.

[38] Cancer Reasearch UK. Cancer incidence for all cancers combined. http://www.cancerresearchuk.org/cancer-info/cancerstats/incidence/all-cancers-combined/#Trends. Accessed 5 Jan 2015.

[39] Yin Z, Brown AE, Hughes G, Nardone A, Gill ON, VC D et al. HIV in the United Kingdom: 2014 Report: data to end 2013. London: Public Health England. 2014.

[40] Siegel R, Ma J, Zou Z, Jemal A (2014). Cancer statistics, 2014. CA Cancer J Clin.

[41] CDC. Estimates of New HIV Infections in the United States, 2006–2009. Atlanta, US: Centres for Disease Control and Prevention. 2011.

[42] Goel R, Shah N, Visaria R, Paciotti GF, Bischof JC (2009). Biodistribution of TNF-alpha-coated gold nanoparticles in an in vivo model system. Nanomedicine (Lond).

[43] Gad SC, Sharp KL, Montgomery C, Payne JD, Goodrich GP (2012). Evaluation of the toxicity of intravenous delivery of auroshell particles (gold-silica nanoshells). Int J Toxicol.

[44] Zhang X-D, Wu D, Shen X, Liu P-X, Fan F-Y, Fan S-J (2012). In vivo renal clearance, biodistribution, toxicity of gold nanoclusters. Biomaterials.

[45] Longmire M, Choyke PL, Kobayashi H (2008). Clearance properties of nano-sized particles and molecules as imaging agents: considerations and caveats. Nanomedicine.

[46] Tudor TL, Townend WK, Cheeseman CR, Edgar JE (2009). An overview of arisings and large-scale treatment technologies for healthcare waste in the United Kingdom. Waste Manage Res.

[47] Waste Generation and Management [database on the Internet]. European Commission. 2013. Available from: http://epp.eurostat.ec.europa.eu/portal/page/portal/waste/waste_generation_management. Accessed: 5 Nov 2013.

[48] DEFRA. Environment Statistics–Key facts. online. London: Department for Environment, Food and Rural Affairs2013 January 2013. Report No.: PB 13671.

[49] USEPA. Progress in Water Quality: An Evaluation of the National Investment in Municipal Wastewater Treatment. online. Washington DC: United States Environmental Protection Agecy2000. Report No.: EPA-832-R-00-008.

[50] British Water. Code of Practice -Flows and Loads 4—Sizing criteria, Treatment Capacity for Sewage Treatment Systems. online. London: British Water 2013. Report No.: BW COP: 18.11/13.

[51] British Water. Codes of Practice—Flows and Loads 3—Sizing criteria, Treatment Capacity for Small Wastewater Treatment Systems. online 2009. Report No.: BW COP: 7.1/09.

[52] McHugh JB (1988). Concentration of gold in natural waters. J Geochem Explor.

[53] Filser J, Arndt D, Baumann J, Geppert M, Hackmann S, Luther EM (2013). Intrinsically green iron oxide nanoparticles? From synthesis via (eco-) toxicology to scenario modelling. Nanoscale.

[54] Eriksson J. Concentrations of 61 trace elements in sewage sludge, farmyard manure, mineral fertiliser, precipitation and in oil and crops. Stockholm, Sweden: Swedish Environmental Protection Agency 2001. Report No.: 5159.

[55] Liu HH, Cohen Y (2014). Multimedia environmental distribution of engineered nanomaterials. Environ Sci Technol.

[56] Praetorius A, Scheringer M, Hungerbühler K (2012). Development of environmental fate models for engineered nanoparticles—A case study of TiO2 nanoparticles in the Rhine river. Environ Sci Technol.

[57] Meesters JAJ, Koelmans AA, Quik JTK, Hendriks AJ, van de Meent D (2014). Multimedia modeling of engineered nanoparticles with SimpleBox4nano: model definition and evaluation. Environ Sci Technol.

[58] ECHA. Guidance on information requirements and chemical safety assessment: Chapter R.16 Environmental exposure estimation. Helsinki, Finland: European Chemicals Agency, 2012.

[59] Dumont E, Johnson AC, Keller VDJ, Williams RJ (2015). Nano silver and nano zinc-oxide in surface waters—Exposure estimation for Europe at high spatial and temporal resolution. Environ Pollut.

[60] Gottschalk F, Ort C, Scholz RW, Nowack B (2011). Engineered nanomaterials in rivers–exposure scenarios for Switzerland at high spatial and temporal resolution. Environ Pollut.

[61] Suarez IJ, Rosal R, Rodriguez A, Ucles A, Fernandez-Alba AR, Hernando MD (2011). Chemical and ecotoxicological assessment of poly(amidoamine) dendrimers in the aquatic environment. TrAC Trends Anal Chem.

[62] ICMM. MERAG: Fact Sheet 03 Effects Assessment: Data Compilation, Selection and Derivation of PNEC Values for the Risk Assessment of Different Environmental Compartments (Water, STP, Soil, Sediment). London: International Council on Mining and Metals, 2007.

[63] Wheeler JR, Grist EPM, Leung KMY, Morritt D, Crane M (2002). Species sensitivity distributions: data and model choice. Mar Pollut Bull.

[64] Stilgoe J, Owen R, Macnaghten P (2013). Developing a framework for responsible innovation. Res Policy.

[65] Ashton D, Hilton M, Thomas KV (2004). Investigating the environmental transport of human pharmaceuticals to streams in the United Kingdom. Sci Total Environ.

[66] Thomas KV, Hilton MJ (2004). The occurrence of selected human pharmaceutical compounds in UK estuaries. Mar Pollut Bull.

[67] Liu J, Lu G, Zhang Z, Bao Y, Liu F, Wu D (2015). Biological effects and bioaccumulation of pharmaceutically active compounds in crucian carp caged near the outfall of a sewage treatment plant. Environ Sci Process Impacts.

[68] Liu J, Lu G, Xie Z, Zhang Z, Li S, Yan Z (2015). Occurrence, bioaccumulation and risk assessment of lipophilic pharmaceutically active compounds in the downstream rivers of sewage treatment plants. Sci Total Environ.

[69] Sanchez W, Sremski W, Piccini B, Palluel O, Maillot-Maréchal E, Betoulle S (2011). Adverse effects in wild fish living downstream from pharmaceutical manufacture discharges. Environ Int.

[70] Shvedova AA, Kagan VE, Fadeel B (2010). Close encounters of the small kind: adverse effects of man-made materials interfacing with the nano-cosmos of biological systems. Annu Rev Pharmacol Toxicol.

[71] Lowry GV, Gregory KB, Apte SC, Lead JR (2012). Transformations of nanomaterials in the environment. Environ Sci Technol.

[72] Linkov I, Satterstrom FK, Steevens J, Ferguson E, Pleus RC (2007). Multi-criteria decision analysis and environmental risk assessment for nanomaterials. J Nanopart Res.

[73] Owen R, Crane M, Grieger K, Handy R, Linkov I, Depledge M. Strategic approaches for the management of environmental risk uncertainties posed by nanomaterials. In: Linkov I, Steevens J (eds) Nanomaterials: Risks and Benefits. NATO Science for Peace and Security Series C: Environmental Security: Springer Netherlands; 2009. p 369–84.

[74] Garner KL, Suh S, Lenihan HS, Keller AA (2015). Species sensitivity distributions for engineered nanomaterials. Environ Sci Technol.

[75] Coll C, Notter D, Gottschalk F, Sun TY, Som C, Nowack B. Probabilistic environmental risk assessment of five nanomaterials (nano-TiO2, nano-Ag, nano-ZnO, CNT, Fullerenes. Nanotoxicology. 2015 (in press). 10.3109/17435390.2015.1073812 26554717

[76] Gottschalk F, Kost E, Nowack B (2013). Engineered nanomaterials in water and soils: a risk quantification based on probabilistic exposure and effect modeling. Environ Toxicol Chem.

[77] Hough R, Noble R, Hitchen G, Hart R, Reddy S, Saunders M (2008). Naturally occurring gold nanoparticles and nanoplates. Geology.

[78] US FDA. Guidance for Industry: Environmental Assessment of Human Drug and Biologics Applications. In: Center for Drug Evaluation and Research (CDER) and Center for Biologics Evaluation and Research (CBER), editor. 1998.

[79] EMA. Guideline on the Environmental Risk Assessment of Medical Products for Human Use. London: European Medicines Agency, USE CFMPFH;2006. Report No.: EMEA/CHMP/SWP/4447/00 corr 1*.

[80] DEFRA. Waste water treatment in the United Kingdom—2012. online. London: Department for Enviroment, Food and Rural Affairs, Department for Enviroment FaRA; 2012. Report No.: PB13811.

[81] USEPA. Clean Watersheds Needs Survey 2008: Report to Congress. online. Washington: United States Environment Protection Agency, Municipal Support Division SMB; 2008. Report No.: EPA-832-R-10-002.

[82] US EPA. Report to Congress on the Impacts and Control of CSOs and SSOs. online. Washington D.C: United States Environmental Protection Agency 2004. Report No.: EPA 833-R-04-001.

[83] ASCE. 2013 Report Card for America’s Infrastructure. online: American Society of Civil Engineers. 2013.

[84] Kaegi R, Voegelin A, Ort C, Sinnet B, Thalmann B, Krismer J (2013). Fate and transformation of silver nanoparticles in urban wastewater systems. Water Res.

[85] Jarvie HP, Al-Obaidi H, King SM, Bowes MJ, Lawrence MJ, Drake AF (2009). Fate of silica nanoparticles in simulated primary wastewater treatment. Environ Sci Technol.

[86] Johnson AC, Jürgens MD, Lawlor AJ, Cisowska I, Williams RJ (2014). Particulate and colloidal silver in sewage effluent and sludge discharged from British wastewater treatment plants. Chemosphere.

[87] Eurostat. Guidance on classification of waste according to EWC-Stat categories:Supplement to the Manual for the Implementation of the Regulation (EC) No 2150/2002 on Waste Statistics. online: Commission of The European Communities: EUROSTAT. 2010.

[88] USEPA. Municipal Solid Waste Generation, Recycling, and Disposal in the United States, Facts and Figures for 2011. online. Washington DC: United States Environmental Protection Agency 2013. Report No.: EPA530-F-13-001.

[89] USEPA. United States Response UNEP Questionnaire for Paragraph 29 study Enclosure 4a April 2010. Revised May 2010. 2010. http://www.unep.org/chemicalsandwaste/Portals/9/Mercury/Documents/para29submissions/USA-Waste%20Incineration_revised%206-1-10.pdf. Accessed 19 Mar 2014.

[90] DEFRA. Incineration of Municipal Solid waste. London: Department for Environment, Food and Rural Affairs. 2013. Report No.: PB13889.

[91] Buffat P, Borel JP (1976). Size effect on the melting temperature of gold particles. Phys Rev A.

[92] Lee J, Lee J, Tanaka T, Mori H (2009). In situ atomic-scale observation of melting point suppression in nanometer-sized gold particles. Nanotechnology.

[93] Dick K, Dhanasekaran T, Zhang Z, Meisel D (2002). Size-dependent melting of silica-encapsulated gold nanoparticles. J Am Chem Soc.

[94] Nanda KK, Maisels A, Kruis FE, Rellinghaus B (2007). Anomalous thermal behavior of gold nanostructures. Europhys Lett (EPL).

[95] Luo W, Su K, Li K, Liao G, Hu N, Jia M (2012). Substrate effect on the melting temperature of gold nanoparticles. J Chem Phys.

[96] Kakumazaki J, Kato T, Sugawara K (2014). Recovery of gold from incinerated sewage sludge ash by chlorination. ACS Sustain Chem Eng.

[97] Honig RE, Kramer DA. Vapor pressure data for the solid and liquid elements. RCA Laboratories, David Sarnoff Research Center; 1969.

[98] Walser T, Limbach LK, Brogioli R, Erismann E, Flamigni L, Hattendorf B et al. Persistence of engineered nanoparticles in a municipal solid-waste incineration plant. Nat Nano. 2012;7(8):520–4. doi:http://www.nature.com/nnano/journal/vaop/ncurrent/abs/nnano.2012.64.html#supplementary-information. 10.1038/nnano.2012.64 22609690

[99] RTI International. Memorandum: Inventory of Hospital/Medical/Infectious Waste Incinerators Potentially Covered by the Proposed Section 111(d)/129 Federal Plan2012. Report No.: EPA Contract No. EP-D-07-019.

[100] AmuthaRani D, Boccaccini AR, Deegan D, Cheeseman CR (2008). Air pollution control residues from waste incineration: current UK situation and assessment of alternative technologies. Waste Manag.

[101] Ørnebjerg H, Franck J, Lamers F, Angotti F, Morin R, Brunner M (2006). Management of bottom ash from WTE plants: an overview of management options and treatment methods.

[102] Mari M, Domingo JL (2010). Toxic emissions from crematories: a review. Environ Int.

[103] Asharani PV, lianwu Y, Gong Z, Valiyaveettil S (2011). Comparison of the toxicity of silver, gold and platinum nanoparticles in developing zebrafish embryos. Nanotoxicology.

[104] Bar-Ilan O, Albrecht RM, Fako VE, Furgeson DY (2009). Toxicity Assessments of Multisized Gold and Silver Nanoparticles in Zebrafish Embryos. Small.

[105] ECHA. Guidance on information requirements and chemical safety assessment:Chapter R.10: Characterisation of dose [concentration]-response for environment: European Chemicals Agency. 2008.

