# Supplementary material for: Probabilistic modelling of prospective environmental concentrations of gold nanoparticles from medical applications as a basis for risk assessment
Source: J Nanobiotechnology. 2015 Dec 22;13:93. doi: 10.1186/s12951-015-0150-0 (PMC4688950; doi:10.1186/s12951-015-0150-0)
Supplement: Supplementary file 1 — 10.1186/s12951-015-0150-0Supporting Information [file 12951_2015_150_MOESM1_ESM.pdf]

Additional file: Supporting Information

Probabilistic modelling of prospective environmental concentrations of gold nanoparticles from medical applications as a basis for risk assessment

Indrani Mahapatra[ǂ], Tian Yin Sun[ǂ], Julian R.A. Clark, Peter J. Dobson, Konrad Hungerbuehler, Richard Owen, Bernd Nowack[ŧ] and Jamie Lead[ŧ]

Number of pages: 43 (AF1-AF43)

Number of figures: 4

Titles of figures:

- **Figure AF-F1** PEC vs pSSD in water with sublethal end points
- **Figure AF-F2** PEC vs pSSD for water and using lethal endpoints
- **Figure AF-F3** PEC vs pSSD in soil with lethal endpoints
- **Figure AF-F4** PEC vs pSSD in water with sublethal end points

Number of tables: 7

Titles of tables:

- **Table AF.T1**: The probabilistic distribution functions of the input parameters used to create the probability mass flow model
- **Table AF.T2:** Prospective per annum amount of Gold nanoparticles in select medical applications (worst case scenario)
- **Table AF.T3.1** Summary of volume or mass of environment compartment – air, water, sediment and soil – as input parameters for the probabilistic mass flow model
- **Table AF.T3.2** Summary of non hazardous household and hazardous healthcare and biological waste
- **Table AF.T3.3** Summary of parameters related to waste water
- **Table AF.T4.1** Data for aquatic toxicity
- **Table AF.T4.2** Data for terrestrial toxicity

[ǂ]: Joint first authors

[ŧ] : Corresponding authors

Table AF.T1: The probabilistic distribution functions of the input parameters used to create the probability mass flow model

Parameters		Probabilistic Distribution Functions(PDFs)		Values	
		UK	US	UK	US
Estimation of Au-NP Consumption (in grams)	Lateral Flow Immunoassay to detect the presence of *Methicillin Resistant and Methicillin Sensitive Staphylococcus aureus* in blood	Triangular	Triangular	0.17-(0.34)-0.51	3-(6)-9
	Test kit for detection and genotyping Warfarin metabolism	Triangular	Triangular	0.18-(0.36)-0.54	1.5-(3)-4.5
	Test kit for detection of single nucleotide polymorphism to detect risk from venous thrombosis	Triangular	Triangular	0.5-(1)-1.5	1.5-(3)-4.5
	OTC test kits to detect pregnancy and ovulation	Uniform	Uniform	3 to 100	20 to 460
	Test kits for qualitative detection of antibodies to HIV-1 and HIV-2 in human serum, plasma and blood	Uniform	Uniform	2 to 80	20 to 830
	Home based in vitro HIV test kits	Triangular	Triangular	10-(20)-30	45-(90)-135
	Test kits to establish viral load In HIV patients	Triangular	Triangular	30-(60)-90	270-(540)-720
	Test kits to diagnose infectious diseases	Triangular	Triangular	35-(70)-105	175-(350)-525
	Nasal decolonization of *Staphylococcus aureus*	Uniform	Uniform	30 to 53300	110 to 164640
	Periodontal disease treatment	Uniform	Uniform	270 to 106560	940 to 365158
	Sensors for diagnosing diseases from breath samples	Uniform	Uniform	0.01 to 1590	0.03 to 4620
	Treatment modality for Cancer : TNF delivery (Can_T1)	Triangular	Triangular	70 -(480) -1100	310- (2020) – 4600
	Treatment modality for Cancer (last line) : TNF delivery (Can_T1_LS)	Triangular	Triangular	210-(420)-630	750-(1500)-2250
	Treatment modality for Cancer: Thermal ablation (Can_T2)	Uniform	Uniform	140290 to 233820	744750 to 1241260
	Treatment modality for Cancer (last line): Thermal ablation (Can T2_L2)	Uniform	Uniform	104710 to 174520	468250 to 780410
	Transbuccal insulin delivery platforms (Dia_T)	Triangular	Triangular	64125-(128250)-192375	420810-(841620)-1262430
TCs after treatment of patients	Can_T1 to wastewater	Fixed data	fixed data	0.65	0.65
	Can_T1 remains in body	Fixed data	fixed data	0.35	0.35
	Can_T2 to wastewater	Fixed data	fixed data	0.15	0.15
	Can_T2 remains in body	Fixed data	fixed data	0.85	0.85
	Dia_T to wastewater	Fixed data	fixed data	1	1

	Can_T1_LS to body	Fixed data	fixed data	1	1
	Can_T2_LS to body	Fixed data	fixed data	1	1
	Body to crematorium	Triangular	Triangular	0.37-(0.74)-1.11	0.19-(0.38)-0.57
	Body to burial	Triangular	Triangular	0.13-(0.26)-0.39	0.31-(0.62)-0.93
**From STP**	Percentage of population not connected to Sewage Treatment Plant	Triangular	Triangular	0.02-(0.04)-0.06	0.13-(0.26)-0.39
	Overflows from STP	Log normal	Uniform	mean=0.161, SD=0.077	0.01 to 0.07
	Leakage from sewerage networks	Uniform	Uniform	0.03 to 0.05	0.05 to 0.06
	STP misconnection	Uniform	--	0.0026 to 0.018	--
	STP removal efficiency	Triangular	Triangular	0.98-(0.99)-1.0	0.98-(0.99)-1.0
	Sludge to Incinerators (WIP)	Triangular	Uniform	0.09-(0.18)-0.27	0.15 to 0.17
	Sludge to Landfill	Triangular	Uniform	0.005-(0.01)-0.015	0.29 to 0.30
	Sludge to soil	Dependent	Dependent		
**Waste**	Hazardous waste to HMCIW Incinerators	Triangular	Triangular	0.2-(0.4)-0.6	0.05-(0.1)-0.15
	Hazardous waste to landfill	Triangular	Triangular	0.3-(0.6)-0.9	0.45-(0.9)-1.35
	Non-hazardous waste to MWI	Triangular	Triangular	0.075-(0.15)-0.225	0.09-(0.18)-0.27
	Non-hazardous waste to landfill	Triangular	Triangular	0.425-(0.85)-1.275	0.41-(0.82)-1.23
**From Waste Incinerator Plant**	Stack emissions from MWI	Triangular	Triangular	0.095-(0.19)-0.285	0.095-(0.19)-0.285
	Bottom-ash from MWI	Triangular	Triangular	0.62-(0.81)-1.0	0.62-(0.81)-1.0
	MWI bottom-ash to landfill	fixed data	fixed data	1	1
	MWI Fly-ash to air	Triangular	Triangular	0.00005-(0.0001)-0.00015	0.00005-(0.0001)-0.00015
	MWI Fly-ash to landfill	Triangular	Triangular	0.99-(0.9999)-1.0	0.99-(0.9999)-1.0
	Au-NP in HMCIWI	Uniform	Uniform	0 to 1	0 to 1
	Gold eliminated from HMCIWI	Uniform	Uniform	0 to 1	0 to 1
	Au-NP from HMCIWI to stack emissions	Fixed data	fixed data	0.19	0.19

	Au-NP from HMCIWI to bottom-ash	Triangular	Triangular	0.81	0.81
	Bottom ash from HMCIWI to landfill	fixed data	fixed data	1	1
	Stack emissions from HMCIWI to wet scrubber	Triangular	Triangular	0.25-(0.5)-0.75	0.25-(0.5)-0.75
	Stack emission from HMCIWI to Dry scrubber and Fabric Filter (APCD)	Triangular	Triangular	0.25-(0.5)-0.75	0.25-(0.5)-0.75
	APCD to landfill	Triangular	Triangular	0.99-(0.9999)-1.0	0.99-(0.9999)-1.0
	APCD to air	Triangular	Triangular	0.00005-(0.0001)-0.00015	0.00005-(0.0001)-0.00015
	HMCIWI wet scrubber to waste water	Uniform	Uniform	0 to 1	0 to 1
	HMCIWI wet scrubber to air	Uniform	Uniform	1 to 0	1 to 0
**TCs between Eco systems**	Air to soil	fixed data	fixed data	0.9866	0.9324
	Air to surface water	fixed data	fixed data	0.0134	0.0676
	Surface water to sediments(S2S)	Worst-case scenario	Worst-case scenario	0 or 1	0 or 1

S2: Estimation of annual Au-NP consumption

Our aim was to identify Au-NP enabled medical applications which are approved, in clinical trials or show promise of translation from pre-clinical models. We have crosschecked our selection of applications used in this study by using corporate websites, company annual reports, press releases, and clinical trials.gov database including US FDA and EMA websites. The subscription database of 'Citeline'[a] and 'Adis R&D Insight' [b] was used between the period of 17 - 21 December 2012, 18 -19 January and 26-27 April 2013. Information obtained from personal communication has also been included to arrive at some generic estimates since the empirical data base is insufficient. United States Patent and Trademark Office's website and 'Patent Buddy' websites were relied upon for finding out related patents to arrive at an estimate of the amount of gold (Au) per test/per patient.

For arriving at population estimates, sources of information include data from the World Health Organization (WHO), www.cancerresearchuk.org, and U.S. federal agencies such as National Institutes of Health (NIH), National Cancer Institute's SEER data base, and the Centers of Disease Control and Prevention (CDC), to name a few has been used. For the UK, data was extracted from the website of the ONS (Office of the National Statistics) and reports from NICE (National Institute of Clinical Excellence) and the NHS (the National Health Services). Where possible and practicable, the most recent data available have been used. Broad assumptions have been used with the intent to come up with best plausible estimates. Attempts have been made to reduce risks due to double counting (Exception: There is double counting of two applications selected for testing of *Staphylococcus aureus*. However, the inclusion of this data does not impact the share of these applications significantly in the total consumption amount. The assumptions are:

- It has been assumed that each product by a company for a particular application serves 100% of the market of the US and UK (i.e. no competition) and all patients, irrespective of socio-economic status etc., have access to these products. For example, when a therapy is in clinical trials for head and neck cancer, we have used the latest publicly available data for number of people diagnosed with head and neck cancer in a particular year and used this data as a prospective population for treatment. Innovative medicines might create excitement with regard to possibility of increasing the life expectancy of a patient; hence we have assumed that all deaths could be prevented if this medicine is used as a last line treatment under the auspices of "expanded access or compassionate use"[c]. Therefore, mortality figures of people suffering from a particular type of cancer were used. We are aware that not all people will have access to these 'trial' drugs and devices, however, our objective is to model high emission worst case scenario and hence we have included these numbers. Various different disease types and stages of cancer have not been taken into consideration. It is assumed that all patients get treated in the same year, since the model (in the current state of development) doesn't allow for time-based-releases.

- Attempts have been made to reduce risks due to double counting (Exception: There is double counting of two applications selected for testing of *Staphylococcus aureus*). However, the inclusion of this data does not impact significantly the share of these applications in the total consumption amount.

---

[a] http://www.citeline.com/

[b] http://www.springer.com/gp/adis/products-services/adisinsight-databases/r-d-insight

[c] http://www.ema.europa.eu/ema/index.jsp?curl=pages/regulation/general/general_content_000293.jsp; http://www.fda.gov/MedicalDevices/DeviceRegulationandGuidance/HowtoMarketYourDevice/InvestigationalDeviceExemptionIDE/ucm051345.htm

- Estimates of health and health care related statistics are based on the most recent data available in the public domain, except for incidences of Venous Thomboembolism for the UK.

- In most cases, dose of the therapeutic agent is used to arrive at estimates and the gold amounts that would be present in drug delivery equipments, containers containing the drug, etc. have not been included in our estimates.

- Census data of the US (2010) and UK (2011) have been used to arrive at the prospective population.

The details of the data and assumptions used to calculate annual consumption of Au-NP from medical applications selected for the study is presented below. The two step approach:

1. Estimate the range of nano gold amount per application
2. Estimate the prospective affected population/total number of tests

Table AF.T2: Prospective per annum amount of Gold nanoparticles in select medical applications (worst case scenario)

Application	Description	Amount per test / intake (unit)	Number of Applications per patient	Possible Population (*UK* and **USA**)	Prospective consumption amount[d]	Refer pages 10 to 20 for specific assumptions to estimate Au amount	End of Life
Diagnostic devices for Pregnancy and Ovulation detection	Lateral flow assay kits to detect the presence of select biomarkers in urine	(2.5 to 8.52)*10^{-7}g	1/year	*12770000*	*3.19 to 10.85*	Refer to **Bullet A**.	Household waste
		(2.5 to 8.52)*10^{-7}g	1/year	**61601000**	**15.40 to 52.36**		
		(2.5 to 8.52)*10^{-7}g	6/year	*19557000*	*29.34 to 136.10*		
		(2.5 to 8.52)*10^{-7}g	6/year	**90732000**	**99.74 to 462.73**		
Diagnostic devices for HIV tests	Rapid Lab based test kits for HIV AIDS	8.52*$10^{-7}$to 3.75*10^{-5}g	Once/year	*2073700*	*1.77 to 77.76*	Refer to **Bullets B.1. and B.2.**	Medical waste
		8.52*$10^{-7}$to 3.75*10^{-5}g	Once/year	**22000000**	**18.74 to 825**		
	HIV *Oral test kits*	8.52*10^{-7}g	Once/year	*20853000*	*17.77*	Refer to **Bullet B.3.**	Household waste
		8.52*10^{-7}g	Once/year	**101777000**	**86.71**		
	Lab based test kits for HIV AIDS	0.000517g	2 times/year	*116000*[e]	*59.97*	Refer to **Bullet G.**	Medical waste
		0.000517g	2 times/year	**1050000**[f]	**542.85**		
Diagnostic device for MRSA/MSSA test	Test is conducted on a positive blood culture report to detect the presence of Methicillin Resistant and Methicillin Sensitive *Staphylococcus aureus* in blood	1.7*10^{-5}g	Once	*20000*	*0.34*	Refer to **Bullet C.**	Medical waste
		1.7*10^{-5}g	Once	**325000**	**5.25**	Refer to **Bullet C.**	
Modality for Infection Prevention	Removal of *Staphylococcus aureus* in the nasal passages to prevent nosocomial	(1.36 to 5.12)*10^{-2}g	2	*439014*	*11976.29 to 44911.1*	Refer to **Bullet D.**	Medical waste
		(1.36 to5.12)*10^{-2}g	2	**1600000**	**43648 to 163680**		
		3.52*10^{-5} to 1.32*10^{-4}g	2	*439014*	*30.90 to 115.899*		
		3.52*10^{-5} to 1.32*10^{-4}g	2	**1600000**	**112.64 to 422.4**		

[d] Unless mentioned, reported unit is gram

[e] Total no. of tests per year

[f] Total no. of tests per year

Application	Description	Amount per test / intake (unit)	Number of Applications per patient	Possible Population (*UK* and **USA**)	Prospective consumption amount[d]	Refer pages 10 to 20 for specific assumptions to estimate Au amount	End of Life
	infection						
Treatment of dental diseases	Treatment of chronic peridontitis, endodontitis, peri-implant diseases	$5.28*10^{-5}$g	1	*5208200*	*274.99*	Refer to **Bullet E.**	Waste water
		$5.28*10^{-5}$g	1	**17847400**	**942.34**		
		$2.05*10^{-2}$g	1	*5208200*	*106559.77*		
		$2.05*10^{-2}$g	1	**17847400**	**365151.80**		
Diagnostic test kits for detecting infectious diseases	Gram positive blood culture tests (Septicaemia)	$5.66*10^{-6}$g	1	*20000*	*0.11*	Refer Bullet G.1.1.	Medical waste
		$5.66*10^{-6}$g	1	**325000**	**1.84**		
	Gram negative blood culture tests	$5.66*10^{-6}$g	1	*75000*	*0.42*	Refer Bullet G.1.2.	
		$5.66*10^{-6}$g	1	**280000**	**1.58**		
	C. difficile test (gram positive bacteria)	$5.66*10^{-6}$g	1	*20851*	*0.12*	Refer Bullet G.1.3.	
		$5.66*10^{-6}$g	1	**370260**	**2.09**		
	Respiratory Virus test	$5.66*10^{-6}$g	1	*12636400*	*71.52*	Refer Bullet G1.4.	
		$5.66*10^{-6}$g	1	**60856000**	**344.44**		
Diagnostic test kit to evaluate hypercoaguable state	Detection of single nucleotide polymorphism (F2/F5) to establish risk from venous thrombosis (VTE)	$5.66*10^{-6}$g	1	*225000*	*1.27*	Refer Bullet G.2.	
		$5.66*10^{-6}$g	1	**550000**	**3.11**		
Diagnostic test kit for genotyping drug metabolism	Genotyping Warfarin metabolism	$5.66*10^{-6}$g	1	*64000*	*0.36*	Refer Bullet G.3.	
		$5.66*10^{-6}$g	1	**550000**	**3.11**		
Sensors for diagnosing diseases from breath samples	Diagnosing of lung, prostate, head and neck cancer, breast, colorectal cancer and Chronic Kidney disease	$2.21*10^{-3}$, $2.21*10^{-6}$, $1.43*10^{-8}$g	1	718401	*0.01 to 1588.71*	Refer Bullet **F.**	Medical waste
		$2.21*10^{-3}$, $2.21*10^{-6}$, $1.43*10^{-8}$g	1	**2087211**	**0.02 to 4615.77**		

Application	Description	Amount per test / intake (unit)	Number of Applications per patient	Possible Population (*UK* and **USA**)	Prospective consumption amount[d]	Refer pages 10 to 20 for specific assumptions to estimate Au amount	End of Life
Treatment for solid tumors (colorectal, pancreas, breast, ocular)	Treatment of cancer by delivery of hrTNF (tumor necrosis factor) bound to gold nanoparticles	95.39% of (95 to 1432 µg)	8 doses for full treatment cycle	*100639*	*0.07-(0.48)-1.10 kg*	Refer **Bullet I.**	Waste water
		95.39% of (95 to 1432 µg)		**421610**	**0.3-(2.024)-4.61 kg**		
	Last line treatment	95.39% of 1432 µg		*36565*	*0.42 kg*		
	Last line treatment	95.39% of 1432 µg		**130640**	**1.50 kg**		Waste water + burial cremation
Treatment for patients diagnosed with head & neck and lung cancer	Photothermal ablation of head and neck cancer and Lung tumor	2793 to 4655 mg[g]	2 doses per treatment cycle	*50230*	*140 to 234 kg*	Refer **Bullet J.**	Waste waster
				**266650**	**744.75 to 1241.25 kg**		
	Last line treatment			*37490*	*104.7 to 174.52* kg		
	Last line treatment			**167650**	**468.246 to 780.41 kg**		Waste water + Burial/cremation
Diabetes Management	Transbuccal Insulin delivery Platform	0.366 mg	One dose every day*365 days	*960,000*	*128.35 kg*	Refer **Bullet K**.	Waste water
				**6300000**	**841.62 kg**		
**UK (total)**					*540 kg*		
**US (total)**					**2700 kg**		

[g] Includes two doses recommended per treatment cycle

A. Test kits to detect pregnancy and ovulation

Seven Pregnancy and ovulation test kits containing colloidal Gold approved by USFDA:

- Atlas Medical
- IND Diagnostics
- Polymed therapeutics
- NewScen Coast Bio-Pharmaceutical
- Tianjin New Bay Bioresearch Co., Ltd.
- Nantong EGENS Biotechnology Co., Ltd.
- Church and Dwight

Assumptions to estimate amount of Au per application

1. Au-NP size = 60-80 nm size[1]
2. Conjugate release pad's width is 15 mm[2]
3. 1µl/mm of conjugate (gold + anti hCG) is used[3]
4. Mass of 60 nm Au-NP/ml = $5.68*10^{-5}$ g/ml [4]
5. Range: 5-15 µl of gold conjugate per test device[5]. Therefore, use 15 µl of conjugate solution per test device: mass of Au = $\mathbf{8.52*10^{-7}}$ g per test device
6. Amount of gold antibody conjugate = 0.03 to 0.25 µg /test device, i.e., $3*10^{-8}$ g per test device and $\mathbf{2.5*10^{-7}}$ **g** per test device [2]

Therefore, we use two estimates of Au per test device for high emission worst case scenario:

1. $2.5*10^{-7}$ g/test device
2. $8.52*10^{-7}$ g/test device

Assumptions for annual total number of tests

- All women in the child bearing (15-44 yrs) age group conduct one pregnancy test per year. The age range of child bearing age has been taken from the reported age range of 15-44 yrs in Table 13 of the report Health, United States, 2011[6]
- 50% women of child bearing age group from (30-44 yrs) conduct 6 ovulation tests per year
- 20 million pregnancy and ovulation tests in the US per year [7]

  – Total female population, aged 15 to 44 yrs, for the US = 61606000[8]
  – Total female population, aged 30-44 yrs, for the US = 30244000[8]
  – Total female population, aged 15 to 44 yrs, for the U.K.= 12777000[9]
  – Total female population, aged 30-44 yrs,, for the U.K. = 6519000[9]

B. Test kits to diagnose HIV

B.1. Four *Rapid* HIV tests approved by USFDA based on colloidal gold

1. Clearview® COMPLETE HIV ½ (Alere)
2. Clearview® HIV 1/2 STAT-PAK (Alere)
3. Uni-Gold Recombigen (TRINITY BIOTECH)
4. OraQuick® ADVANCE Rapid HIV-1/2 (Orasure technologies)

CE marked (European Union)

1. Genie™ Fast HIV ½ (Bio-Rad)

Assumptions to estimate amount of Au per application

1. Particle size: 5-50 nm [10]
2. Mass of Au-NP/ml = $5.68*10^{-5}$ g/ml [4]
3. Gold conjugate solution = 10 µl/test strip [11]
4. Gold conjugate solution = 15 µl/test strip [5]

We use 15 ul/test strip = $8.52*10^{-7}$ g Au/test strip

B.2. Colloidal Gold based laboratory based HIV tests [12]

Assumptions to estimate amount of Au per application

1. Au-NP size = 80 nm[1]
2. 10 ml vial[12]
3. Per vial caters to 15 tests[12]. So, amount of gold solution per test is 0.66 ml
4. Mass of Au/ml = $5.68*10^{-5}$ g/ml[4]
5. Mass concentration of Au (80 nm) per 0.66 ml or per test device = $3.75*10^{-5}$ g

Assumptions for annual total number of tests

Number of HIV tests conducted per year in the US= 16-22 millions[13]

To estimate for high emission scenario, we use the higher value = 22 million tests for the US

For the UK

- All people who attended Sexual Health Clinics are tested for HIV AIDS in 2013 = 1373700[14]
- Total no. of women tested under antenatal screening program in 2013 = 700000[14]

Therefore, total number of HIV tests for the UK in 2013 = 2073700

B.3. Colloidal Gold based HIV home based test kits

Approved by US FDA on 3 July 2012[15]

Assumptions to estimate amount of Au per application

1. Au-NP size = 60 nm[1]
2. 15 µl/test device = $8.52*10^{-7}$ g/test strip[5]

Assumptions for annual total number of tests

Since this is a home based test based on oral fluids, we assume 50% of people **from age 15 to 64 years** conduct one home based HIV test per year, though legally the self-testing kit is to be sold to population aged 17 years or more, we have used 15-64 yrs because of the class intervals provided in the population tables.

- Population in the age group of 15 to 64 yrs for the US (Year 2010) = 203 554 000[8]
- Population in the age group of 15 to 64 yrs for the UK (Year 2011) = 41 706 000[9]

C. Lateral flow Immunoassay test for detection of Methicillin Resistant and Methicillin Sensitive Staphylococcus aureus in blood

Assumptions to estimate amount of Au per application

1. Au-NP size = 80 nm (20-80 nm for Lateral Flow Devices and Conjugates)
2. Mass gold /ml = $5.69*10^{-5}$ g/ml [4]
3. 15 µl of gold conjugate solution per strip[5]
4. **Two** test kits per test[16]. Therefore, 30 µl of gold conjugate per test, i.e., 0.03ml = $1.7*10^{-5}$ g of Au per test device

Assumptions for annual total number of tests

US:

No. of discharges with septicaemia = 1665400. Around 15% (approx 250000) of the above discharges were diagnosed to be due to gram positive bacteria[17]

50% of patients suffering from septicaemia, the bacteria is unspecified. And, 15% have bacteria present in blood, but without the response. Keeping these factors into consideration, assume 30% more tests to be done.[17]

Therefore**, total no. of tests** = 25000 + 30% of 250000. **ca.325000**

UK:

No. of MSSA and MRSA reports in England (above 2 years of age) year 2013 = ca. 10000 [18]

Population for England above 4 years is ca. 50 million[9].

Total population over 4 yrs for UK = ca. 60 million[9]

So, for the UK = estimated number of MRSA and MSSA cases is 12000 (above 4 years of age) approx. = assume 15000 for all age groups.

Therefore, **total no. of tests** = 15000 + 30% more tests = 15000 + 4500 = ca. 20000

D. Nasal decolonization of Staphylococcus aureus

Assumptions to estimate amount of Au per application

1. Au-NP size = 2 and 15 nm[19]
2. One vial = 1.5 ml, 54 vials in a pack[20]
3. Two treatments per patient[21]
4. 2nm Au-NP has ca. 270 atoms (M_{Au} = 53000 Da)[22]
5. Mass of one Au-NP of 2 nm = 53000 dalton = $8.8*10^{-20}$ g[22]
6. Particle mass of 15 nm Au-NP= $3.41*10^{-17}$ g[4]
7. Total particles in 1 ml = ($1*10^{13}$ to $1 * 10^{15}$)[19]. Use: $1 * 10^{15}$ particles /ml. Therefore, no. of particles in 1.5 ml =$1.5*10^{15}$
8. 1 drop = approx. 0.05 ml
9. 8 drops per patient= 0.4 ml per patient. Therefore, no. of particles in 0.4 ml = $0.4*10^{15}$.

Therefore, we use two estimates of Au per treatment for high emission scenario based on assumed particle size of 2 nm and 15 nm and volume of 0.4 ml and 1.5 ml:

- Amount per treatment (2 nm size)= $3.52*10^{-5}$ g (0.4 ml) to $1.32*10^{-4}$ g (1.5 ml)
- Amount per treatment (15 nm size) = $1.36*10^{-2}$ g (0.4 ml) to $5.12 *10^{-2}$ g (1.5 ml)

Assumptions for annual total number of tests

10-40% of population as outpatients or upon admission have nasal colonisation of S. aureus[23]

ca. 2% - 5% is the rate of Surgical Site Infections[24]

We assume screening/treatment of 10% of the all surgical procedures (inpatients), because people with surgical procedures are at risk of contracting MRSA

US – ca. 16 million surgical procedures conducted (2010) (short stay discharges with procedures from non federal hospitals)[25]

Therefore, 10% of 16 million gives are the prospective number of patients treated = 1600000 for the US

UK – Sum of Scotland, England, Wales and Northern Ireland =10% of (0.25 million + 3749225+0.25 million +0.18 million) = 439014 patients treated

i. **Scotland:** Total main procedures/operations and inpatients stay greater than zero days for year 2011-2012 is 242518 = ca. 0.25 million[26]

ii. **England:** Total main procedures (minus drug therapy and diagnostic) = 8520965 (2011-2012). Inpatients = ca. 44% of 8520965 = 3749225[27]

iii. **Wales:** Total inpatients for the year 2011= 226911 = ca. 0.25 million[28]

iv. **Northern Ireland:** Total main procedures for the year 2011 -12 = 350651[29], 48.9% [30] were inpatients = 48.9% *350651 = 171,483 = ca. 0.18 million

E. Periodontal disease treatment

Assumptions to estimate amount of Au per application

1. Au-NP size = 2nm and 15 nm[19]
2. Mass of 2 nm Au-NP = $8.8*10^{-20}$ g[4]
3. Mass of 15 nm Au-NP = $3.41*10^{-17}$ g[4]
4. Application dose = 0.2 ml of solution per pocket[31]

5. Total dose: 0.6 ml per patient (3 teeth treated per patient)
6. No. of Au-NPs/ml = ($1*10^{15}$)[19]

Therefore, we use two estimates of amount of Au per patient based on particle size of 2nm and 15 nm:

- 2 nm Au-NP size = $5.28*10^{-5}$ g
- 15 nm Au-NP size = $2.05*10^{-2}$ g

<u>Assumptions for annual total number of tests</u>

Background data to arrive the assumption for total number of tests

US:

Definitions [32]

i. Severe periodontitis: Two or more interproximal (IP) sites in different teeth having>= 6 mm Attachment loss AND 1 or more IP site >= 5 mm pocket depth
ii. Moderate periodontitis: Two or more I.P. sites >= 4 mm attachment loss OR two or more I.P. sites >= 5 mm pocket depth

- 47.2% of adults over 30 yrs of age in the United States have some form of periodontal disease[32]
- 8.5% of the adult population (30 years or more) in the U.S suffer from severe periodontitis
- 30% of the adult U.S. Population suffer from moderate periodontitis

U.K.:

- 45% of all dentate (at least 1 teeth) adults, age 16 yrs or more, have pocketing depth of 4 mm or more[33]
- 8% of all dentate adults, greater than 16 yrs of age, pocket depth >6 mm[33]
- 8% of all dentate adults, greater than 16 yrs of age, loss of attachment > 5.5 mm and 5% of all dentate adults aged 16 yrs or more = Pocketing depths > 5.5 mm[34]
- Percentage of total finished admission episodes dealing with periodontitis and gingivitis = 9%[35]

10-15% of world adult population (greater than 15 yrs of age) -severe periodontitis, i.e. Community Periodontal Index = 4, Pocket depth of >= 6 mm[36]

<u>Assumptions for annual total number of tests</u>

- 10% of the population of the U.S. above 30 yrs of age will seek treatment for periodontitis
- 10% of the population of the U.K. above 15 yrs of age will seek per seek periodontitis treatment
- Total population of the US above 30 years = 178474000[8]
- Total Population of the UK above 15 years of age = 52082000[9]

F. Sensors for diagnosing diseases from breath samples

Assumptions to estimate amount of Au per application

1. Au-NP Size = 5nm; an array of monolayer capped spherical Au-NP.
2. Mass of 5 nm Au-NP = $1.26*10^{-18}$ g [4]
3. One drop as 180 pl[37]
4. Or , 1 drop as 0.05µl[38]
5. Or, 1 drop as 0.05 ml
6. 9 sensors with 9 different surface cappings [39]
7. The sensor consists of 10 pairs of circular interdigitated (IDE) gold electrodes of 3 mm diameter and 20 µm electrode width and 20 µm electrode gap (Peng et.al, 2009).
8. 10 drops per circular IDE [40-43]
9. Disposal of sensors array every 10 tests[8].

Therefore,

- 9 sensors*0.05 ml per drop *10 drops =4.5 ml/per sensor array
- 9 sensors*0.05µl per drop *10 drops= 4.5 µl/ = 0.0045ml
- 9 sensors*180 pl*10 drops =9*1.8*10^-6*10=0.000162 ml/sensor

25 ml of 31.5 mM $HAuCL_4$ solution = 0.0315 moles/litre of $HAuCL_4$ solution[39]

Moles of $HAuCL_4$ solution in 25 ml = $7.875*10^{-4}$ moles/L[44]

No. of atoms in a 5 nm particle = (Radius of Au-NP divided by radius of one atom of Gold NP) = $(5/0.137)^3$ = 48612 atoms of Au per NP.

No of nanoparticles formed = 4.74*10^20 atoms of Au divided by No. of atoms of Au per NP

= 48612 = $9.75*10^{15}$ Au-NP

Therefore 25 ml of 31.5 mM of $HAuCl_4$ forms = $9.75*10^{15}$ Au-NP

10. Number and Mass of Au-NP in different volumes:

- Volume 4.5 ml = $1.76*10^{15}$ Au-NP; Mass of Au = $1.76*10^{15}$ * $1.26*10^{-18}$ = $2.21*10^{-3}$ g
- Volume 0.0045ml = $1.75*10^{12}$ Au-NP; Mass of Au =$1.75*10^{12}$ Au-NP * $1.26*10^{-18}$ g =$2.21*10^{-6}$ g
- Volume 0.000162 ml = $1.26*10^{8}$ Au-NP; Mass of Au = $1.26*10^{8}$ Au-NP * $1.26*10^{-18}$ g =$1.59*10^{-10}$ g

Assumptions for annual total number of tests

Type of cancer	US (estimated cases in 2014)[45]	UK (cases for 2011) http://www.cancerresearchuk.org/
Lung	224210	43463
Colorectal	136830	41581
Head and neck cancer	42440	6767
Prostate	233000	41736
Breast	235630	50285
**Total**	**872110**	**183832**

[8] Disposal of sensor after every 100 tests for asthma diagnosis http://www.niox.com/en/ordering/)

Chronic Kidney disease (CKD):

US = 20 million [46]

UK = Range of CKD 44607 to 7291480 = ca 7 million (Roderick et al., 2011).

G. Tests To Diagnose Disease Conditions

G.1. Infectious Disease

<u>Assumptions to estimate amount of Au per application</u>

1. Au-NP size = 13-20 nm [47]; assume Au-NP size = 20 nm
2. Volume per test cartridge: 0.1 ml, i.e., ca. 2 drops
3. Mass of gold per ml = $5.66*10^{-5}$ g[4]; mass of gold in 0.1 ml or mass of Au per application= $5.66*10^{-6}$ g

<u>Assumptions for annual total number of tests</u>

G.1.1 Septicaemia (Gram positive blood culture test)

Refer to details in Page 12 for assumptions for annual number of tests.
US = 325000
UK = 20000

G.1.2 Gram Negative Blood culture test

US = No. of discharges with septicaemia = 1665400[17]

No. of discharges with gram negative bacterial incidences = 215000[17]

Assume, 30% more tests are done. Total no. of tests = 215000 + 30% of 215000 = 280000

Total no. of E-coli infections in England = 33336 for year 2013[18]

Assume 50000 for the UK for all gram negative infections

Assume, 30% more tests are done. Therefore, total no. of tests for the UK= 30% of 50000 +50000 = 75000.

G.1.3. *C.* difficile infections (CDI)

336, 600 hospitalizations that involved CDI in 2009[48]

Assume 10% more diagnostic tests have been performed

So, no. of tests/year for the US = 10% of 336600 +336600 = 370260

For England, reported cases is 13756 for the year 2013[18]

To estimate reported cases for CD infections for the UK, using the rate of 30 per 100000 of population = 18955[18]

Assume 10% more tests conducted

No. of tests done per year for the UK = 20851

G.1.4 Respiratory Virus

USA = 5 to 20% of the population every year [49]

Assume, all people having flu like symptoms are tested for respiratory virus.

Incidences of flu = 20% of total population of the US = 60856000

UK = Same assumption as that for the US, i.e. 20% of population

Flu season = October to May[49]

G.2. Test kit for detection of single nucleotide polymorphism (F2/F5) to establish risk from venous thrombosis (VTE)

<u>Assumptions to estimate amount of Au per application</u>

1. Au-NP size = 13-20 nm [47]; assume Au-NP size = 20 nm
2. Volume per test cartridge: 0.1 ml, i.e., ca. 2 drops
3. Mass of gold per ml = $5.66*10^{-5}$ g[4]; mass of gold in 0.1 ml or mass of Au per application = $5.66*10^{-6}$ g

<u>Assumptions for annual total number of tests</u>

1. Prevalence of Factor V Leiden in European Whites = 3-15%[50]
2. Prevalence of Factor V Leiden in UK = 8.8%[50]
3. Prevalence of Factor V Leiden in Unites States, white population = 5.2%[50]

Assume, 8% of the white population will carry Factor V gene mutation

US white population = 223553265[9] = 8% of 223553265 = 17884261

White population for England and Wales = 54809000[51] = 8% of 54809000 = 4384720 = approx. 4400000

Estimated annual average of hospitalizations with VTE (≥18 years in the United States) = 547596 among those aged ≥18 years in the United States[52]

547596 hospitalisations shows 3% of the white population of the US who might carry one of the risk factors for VTE are hospitalised in a given year.

Therefore, we assume 5% of the white population of the US and UK gets the genetic test done.

5% of 4400000 for the UK = approx. 225000

G.3. Test kit for detection and genotyping Warfarin metabolism

<u>Assumptions to estimate amount of Au per application</u>

1. Au-NP size = 13-20 nm [47]; assume Au-NP size = 20 nm

[9] http://www.infoplease.com/us/statistics/us-population-by-race.html

2. Volume per test cartridge: 0.1 ml, i.e., ca. 2 drops
3. Mass of gold per ml = $5.66*10^{-5}$ g[4]; mass of gold in 0.1 ml or mass of Au per application = $5.66*10^{-6}$ g

Assumptions for annual total number of tests

To establish Warfarin dosages in patients diagnosed with VTE, we assume all hospitalisations/diagnosis with VTE are advised the genetic test for Warfarin metabolism to establish sensitivity to Warfarin and rate of metabolism.

UK, 64000 Finished Consultant Episodes of VTE for the year 2004-05 [53]

For the US, VTE diagnosis = 547596 = approx. 550000[52]

H. Test To Establish Viral Load In HIV Patients

Assumptions to estimate amount of Au per application

1. Au-NP size = 80 nm [54]
2. One polypropylene vial for 20 tests [55]
3. Assume each vial is 2.5 ml. Therefore, 0.125 ml per test.
4. No. of particles per ml = $8*10^{11}$ [54]
5. Mass of one gold NP of 80 nm size = $5.17*10^{-15}$ g[4]
6. Amount of Au in 0.125 ml = 0.000517 g. Therefore, amount of Au per test device = **0.000517 g**

The test is to manage disease progression (start ARV therapy or change drugs when the disease becomes drug resistant).

Population assumptions for annual total consumption

US:

- HIV prevalence (year end 2010) = 872990[56]
- HIV incidence (new diagnosis) is = ca. 50000 every year[56]
- Stage 3 HIV prevalence = ca. 500000 (end of 2010)[56]
- 500,000/872,990 = ca. 60% of people are in Stage 3 of total people living with HIV/AIDS
- Assume people with Stage 3 HIV infection and are on regular Anti-retroviral therapy
- Assume device is used once every 6 months to check their CD4 count. Therefore, Total tests done for patients living with Stage 3 HIV per year =500000 * 2 = 1 million [57]
- Total tests per year = Newly diagnosed + test for HIV stage 3 = 1 million + 50000 = **1050000**

UK:

- Newly diagnosed = 6000[14]
- 107,800 people are living with *known* HIV infection. Assume 50% of the people living with known HIV infection are late stage = 53900 = approx. 55000[14]
- Total tests done for patients living with Stage 3 HIV per year = 55000*2 = 0.11 million =**116000**
- Total tests = Newly diagnosed + test for HIV stage 3 = 0.11 million + 50000

I. Treatment modality for Cancer : TNF delivery

Assumptions to estimate amount of Au per application

1. Au-NP size = 30-34 nm[58]
2. Total dose range of CYT-6091 = 90 ± 5 to 1208 ± 214 µg; therefore, use dose = 95µg to 1432 µg[59]
3. SH- PEG = 20 kDa [60]
4. TNF monomer = 17 kDa. Assume = 20 kDa[58]
5. One Au-NP has 400 TNF molecules bound to it[58]
6. Since the available literature doesn't inform of the number of PEG on one Au-NP[61]. Assume, both SH-PEG and rhTNF are bound to the Au-NP and they do not cross-link with each other.
7. Mass of 1 Au-NP of size 30 nm = $2.73*10^{-16}$ g [4]
8. Mass of 400 TNFs = 400* 20 kDa = 400* $3.32*10^{-20}$ g = $1.32*10^{-17}$ g (Conversion from Da to grams)
9. Ratio=Au-NP: TNF = (2.73*10^-16 /$1.32*10^{-17}$) = 20.76 : 1. Thus, percentage weight of gold is (20.76/21.76)*100 = 95.39%
10. No. of doses per treatment cycle (high dose) = 8 ; 4 courses where 1 course = 2 doses)[59]

Amount of Au per patient:

- Estimates of range of Au per patient: 95.39% of (95*8) µg to 95.39% of (1432*8) µg
- Estimate of average amount of Au per patient = 95.39% of (4801 ug)

<u>Population assumptions for annual total consumption</u>

Type of enrolled patients in clinical trial phase I [59]:

1. Ocular melanoma
2. Adenocarcinoma of the colon and pancreas
3. Ductal carcinoma of breast
4. Carcinoma of rectum

Combine adenocarcinoma of the colon and carcinoma of rectum as colorectal cancer or bowel cancer.

**Type of cancer**	**US**		**UK**	
	Estimated cases in 2014[45]	*Estimated deaths for 2014[45]*	*Diagnosed Cases for 2011*	*Deaths in 2012*
Colorectal	136830	50310	41581[62]	16187[62]
Pancreatic	46420	39590	8773[63]	8662[63]
Breast	235630	40430	50285[64]	11716[64]
Ocular	2730	310	No data	No data
Total	421610	100639	130640	36565

J. Treatment modality for Cancer: Thermal ablation

<u>Assumptions to estimate amount of Au per application</u>

1. Dosage= 21 to 35 mg/kg body [65]
2. Two infusions is the expected clinical dose [65]
3. Average body weight = 70 kg
4. 95% of the weight of Auroshells is gold weight [65] Auroshells: 155 nm in diameter (120 nm diameter is the silica core) with a coating of polyethylene glycol 5000.

Estimates of Amount of Au per patient

- 95% of (21*70*2) =2793mg
- 95% of (35*70*2) = 4655mg

Population assumptions for annual total consumption

Type of cancer	US		UK	
	Estimated cases in 2014[45]	Estimated deaths for 2014[45]	Diagnosed Cases for 2011	Deaths in 2012
Lung cancer[10]	224210	159260	43463[66]	35371[66]
Head and Neck Cancer	42440	8390	6767 (oral cancer)[67]	2119 (oral cancer)[67]
**Total**	**266650**	**167650**	**50230**	**37490**

K. Transbuccal Insulin Delivery Platforms

Assumptions to estimate amount of Au per application

1. Au-NP size = 3.5 nm = 102 atoms of Au [68]
2. Mass of 3.5 nm Au-NP = 102 atoms *196.96 g/ mol = $3.33*10^{-20}$ g
3. 1 IU of insulin = 0.0385 mg [69]
4. Average body weight = 70 kg
5. Total daily insulin intake dose = 0.55 IU/kg of body weight[11] (without giving consideration to insulin resistance, other oral medications, etc.) = 0.55*70 = 38.5
6. Molecular weight of insulin monomer = 5808 Da [69] = ca 5808 g/mol
7. No. of Insulin monomer required per day = 38.5 * 0.0385 mg (Mass of Insulin) = 1.48mg of Insulin/day = $2.5*10^{-7}$ moles of Insulin = $1.5*10^{17}$ molecules of Insulin
8. Binding of Insulin to NP is in the ratio of 14:1 (14 insulin monomer) [68]
9. No. of Au-NP required for binding $1.5*10^{17}$ molecules of Insulin = $1.07*10^{16}$
10. Gold concentration = 4.037 mg of Au/ml =1.21 X 10^{17} Au-NP/ml[68]
11. Mass of $1.07X10^{16}$ Au-NP = 0.366 mg of Au.

Therefore, Amount of Au per day per patient = **0.366 mg**

Population assumptions for annual total consumption

- Total diagnosed diabetic population in the US of all age groups (all ages, 2012) =21 million[70]
- Total diagnosed adults (greater than 18 years) take insulin = 6 million, i.e. 28% of the diagnosed adult population[70]
- People with diagnosed diabetes (20 years and less) = 215000[70]

Therefore, assume 30% of the diagnosed population of all age groups take insulin = **6.3 million**

UK =3.2 million people have been diagnosed with diabetes (2013) [71]

[10] http://www.nanospectra.com/clinicians/trialinfo.html: The clinical trials include metastatic lung cancer and refractory head and neck cancer

[11] http://dtc.ucsf.edu/types-of-diabetes/type1/treatment-of-type-1-diabetes/medications-and-therapies/type-1-insulin-therapy/calculating-insulin-dose/

Also, assume 30% of UK’s diabetic patients will take insulin (as derived from the American numbers) = 30% of 3.2 million = **960000**

Table AF.T3.1 Summary of volume or mass of environment compartment – air, water, sediment and soil – as input parameters for the probabilistic mass flow model. The Comments column provides the values used to calculate the mass/volume. The mass of soil and sediment compartment has been arrived at by multiplying the area, the mixing depth and the density. The area of natural and urban soils has been calculated by subtracting the area occupied by agricultural soils and other soils. Littoral sediments (beaches and intertidal mud flats and salt marshes) have been included for the UK as it represents a key ecosystem of the UK

Compartments	Countries	Formula/ Calculation	Mass/Volume	Unit	Comments
**Sludge treated soils**	UK	$1.65*10^9*0.2*1.5*10^3$	4.95E+11	kg	• $1.65*10^9$ m^2: total sludge treated agricultural land area in the UK[72]<br>• 0.2 m: the depth of agricultural soil[73]<br>• 408,139,000 acres is the total cropland/arable land in the US[12]= ca.$1.65*10^{12}$ m^2 [74, 75]<br>• Total area of sludge treated soil the US: 1%[76] of arable land= $1.65*10^{10}$ m^2<br>• $1.5*10^3$ kg/m^3: the density of dry soil [77, 78]
	US	$1.65*10^{10}*0.2*1.5*10^3$	4.95E+12	kg	
**Surface water**	UK	$3.25*10^9*3*1000$ $*(365/40)$	8.90E+13	litre	• $3.25*10^9$ m^2 : the total freshwater area in the UK [79]<br>• 3 m: the mixing depth of surface water<br>• 1000: the conversion from m^3 to litre<br>• 86,409 sq. Miles: the area of Inland water[80]<br>• 59,959 sq. Miles: the area of Great Lakes [80]<br>• $2.59*10^6$: the conversion factor from sq. mile to m^2<br>• 40:ENM residence time in the system[81]<br>• 365 days: 1 year
	US	$(86409+59959)*2.59*10^6*3*1000$ $*(365/40)$	1.04E+16	litre	
**Surface water Sediments**	UK	$(3.25+2.59)*10^9*0.03*0.82*10^3$	1.44E+11	kg	• $3.25*10^9$ m^2: the total freshwater area[79]<br>• $2.59*10^9$ m^2: the total littoral sediment area[79]<br>• 0.03 m: the depth of sediment<br>• $0.82*10^3$ kg/m^3: the bulk density of dry sediments[78],[82]<br>• 86,409+59,959 sq. Miles: the surface water area in the US [80]<br>• $2.59*10^6$: the conversion factor from sq. mile to m^2
	US	$(86409+59959)*2.59*10^6*0.03$ $*0.82*10^3$	9.33E+12	kg	
**STP Effluent**	UK	$11*10^9*365$	4.02E+12	litre	• 11 billion L: the amount of wastewater collected/day in the UK[83]<br>• 365 days: 1 year<br>• $3.09*10^8$: US population (2010)[8]<br>• 165 US gallons wastewater is assumed per capita per day[84]<br>• 3.785: gallons to litres
	US	$3.09*10^8*165*3.785*365$	7.04E+13	litre	
**STP sludge**	UK		1.41E+09	kg	• $1.41*10^9$ kg : STP sludge (dry weight) generated in the UK [83]<br>• $7.18*10^9$ tons: Biosolids[13] generated in the U.S. [76, 85]<br>• 0.9072: Short Ton to metric Ton
	US		6.5 E+09	kg	

[12] 1 acre = 0.004046 km^2

[13] In the US, treated sewage sludge is termed as biosolids.

Table AF.T3.2 Summary of non hazardous household and hazardous healthcare and biological waste

Compartments	Countries	Formula/ Calculation	Mass/ Volume	Unit	Comments
**Hazardous healthcare and biological (H&B) waste treated by incineration**	UK		$1.4*10^{8}$	kg	• 144,000 tonnes of H&B waste incinerated in year 2008,i.e., 40% of hazardous H&B waste generated[86]<br>• 146,502 tons: Estimated throughput of 54 medical waste incinerators in year 2011[87]
	US	146502*0.9072	$1.1*10^{8}$	kg	
**Hazardous H&B waste sent to landfill**	UK	Total hazardous H&B waste generated – waste incinerated	$2.1*10^{8}$	kg	• 350,000 tonnes: Total hazardous H&B generated in the year 2008[86]
	US	Total hazardous H&B waste generated – waste incinerated	$1.2*10^{9}$	kg	• 5.9 million tons: Total H&B waste generated per year[88]<br>• 24%[89] of 5.9 million tons: Total hazardous waste generated per year
**Non hazardous household waste treated by incineration**	UK	15%*19 million tonnes	$2.8*10^{9}$	kg	• 19,354,616 tonnes of household and similar waste generated in the year 2010[90]<br>• 15% of waste treated (2008)[91]<br>• Assume waste generated = waste treated<br>• 29 million tons: Incineration with energy recovery[92]
	US		$2.6*10^{10}$	kg	
**Non hazardous household waste sent to landfill**	UK	85%*19 million tonnes	$1.6*10^{10}$	kg	• 85% of waste treated is landfilled (2008)<br>• 134 million tons: Municipal waste Landfilled in Year 2011[92]
	US		$1.2*10^{11}$	kg	

Table AF.T3.3 Summary of parameters related to waste water

Parameters	Countries	Values	Comments
**Connection rate to STP**	UK	0.96	• 96%: Percentage of population connected to STP[83]
	US	0.74	• 74%: Percentage of population connected to centralised STP[93]
**Misconnection of STP pipes**	UK	0.0026 to 0.018	• Range estimated from [94, 95 and Personal communication with Bryan Ellis]. Please see explanation in Notes
	US	*No data available*	• None
**Leakage of STP pipes**	UK	0.03 to 0.05 of effluent collected	• Range estimated from [96-98, 83]. Please see explanation in Notes
	US	0.05 to 0.06 of effluent collected	• Range estimated from [99, 98]
**Overflows**	UK	Mean= 0.161, Sd=0.079	• 16.1% of dry weather flow (std dev =7.9% with lognormal distribution) (personal communication with Constantino Carlos)
	US	0.01 to 0.07	• Range estimated from [93, 100]. Please see explanation in Notes

NOTES for AF.T3.1, AF.T3.2, AF.T3.3

1. **Dry weather flow**

Dry weather flow = Population served * per capita water output + Infiltration + trade effluent

Total Population (2010) census = 63,182,000

Population served by WWTP = 96%

Population served = 96% * 6318200 = 60654720

Per capita waste water output = 0.15 m^3/ day [101]

Total population output = 60654720*0.15 m^3 * 365 days = 3.32 billion m^3

Per capita industrial output =0.028 m^3/day [102]

Infiltration = 25% of population WW load = 25% * 3.32E+09 = 8.30E+08 m^3 = 830 million m^3 [102, 103]

Trade flow per year = 0.028 m^3/day * 60654720 * 365 = 6.20E+08 $m^{3\,=}$ 620 million m^3

Total days in a year =365

DWF/year = 4.77E+09 m^3 = 4.77 billion m^3 (nearly same as waste water collected - 4.02 billion m^3)

Storm tank discharges = 16.1% of dry weather flow (personal communication with Constantino Carlos)

2. **Misconnections Volume (UK)**

- Total no. of unshared dwellings in 2011(whole house or bungalow) [95] = 20514994
- Misconnection rate = 1 to 7% and national average = 3% [94]
- 1% to 7% of 20514994 = 205150 to 1436050
- 138 litres WW per day per household discharged due to misconnections[94] (personal communication with Bryan Ellis)
- Misconnection volume = 0.138*365*205150 to 0.138*365*1436050 =10333402 to 72333817 m^3
- Volume percentage of WW discharged due to misconnections= 10333402/4.02E+09 72333817/4.02E+09= 0.26% to 1.8 %

3. **Exfiltration/leakage for the UK**

- Exfiltration rate: 0.0014 l/s/km or 2.8% of DWF for the city of Dresden [cited in 97]
- Exfiltration = 3% of total average annual flow for Thames region [98]
- 5-20% leakage rate for gravity sewers above water table. 5% is the lower value used in various studies [mentioned in 98]; Other studies give very high exfiltration rate [see 96, 104] (see ref. 27 summary and ref. 35 for recent study for Doncaster, UK)
- Range used for our study = 3 to 5% of effluent volume
- Sewer length in the UK = 347,000 km [83]

- Effluent volume (2011)= 4.05 billion m^3 [83]

4. **Overflows/intermittent discharges for the US**
   - Discharges from decentralized water treatment systems due to failures: 66 to 144 billion Gallons[93]
   - Discharges from sanitary sewerage = 900 billion gallons[100]
   - Total overflows = 144+900 = 1,044 billion gallons
   - Total centralized + decentralized effluent= 5.21E+10 + 5.96E+09 = 5.81E+10 m^3
   - Percentage = 3.95E+09 / 5.80E+10 = 6.8% = ca. 7% (higher estimate because the infrastructure report card rates the waste water treatment infrastructure status of US to be 'D', i.e., poor and at risk[100]
   - Conservative estimate from USEPA's Report to Congress (2008): 10 billion gallons from Sewer overflows and 160 billion gallons from Combined Sewer Overflows = 1.1% or ca. 1% of total effluent volume
   - Range = 1 to 7%

5. **Sludge distribution**

UK – [83]

**Total sludge generated/annum**	1412836 (tonnes dry weight)	Percentage
Land application	0.791	79.10%
Incineration	0.184	18.40%
Landfill	0.006	0.60%
Other disposal	0.019	1.90%

US [85, 105]

Total biosolids from Treatment works treating domestic sewage	7180000 dry tons	S	**Range**
**Conversion to metric tonnes**	6513586.43		6.5 to 9.1 million tonnes
Agriculture /farmlands	2651029.68	41%	41% to 45%
Incineration	967267.59	15%	15% to 17%
Landfill+Monofill	1963846.31	30%	29% to 30%
Class A exceptional quality as biosolids or heat dried pellet fertilizer/Compost - silviculture, horticulture, gardens, etc.	788143.96	12%	9% to 14%
Forest land and reclamation, other beneficial uses	143298.90	2%	

6. **Cremation of bodies:**

   UK : Cremation – 74% for year 2012[106]

   US: Cremation - 38% for year 2012[107]

7. **Hospital waste estimates from various sources for the US:**

- More than 4 billion pounds of waste disposed in 2007[108]
  1 pound = 0.45 kg
  Year 2007 = 4*0.45*109 = **1.8 million metric tonnes**

- 2 million tons/year[109] = 2 * 0.9072= 1.8 million tones (if 7000 tons of waste per day = 2.32 million tonnes of waste per year)
  1 ton = 0.9072 tonnes
  1 year = 365 days
  Hospital waste generation range = **1.8 to 2.32 million tonnes**

- 13-15 pounds of waste/patient/day = 5.85 to 6 kg/patient/day[110]

  Use = 6 kg/patient/day

  Total waste = waste/patient/day * total no. of discharges in a year * average length of stay in a hospital

  Total no. of discharges (in non-federal, short stay hospital) in 2008-2009 = 35908000 (Table 104)[6]

  Average length of stay (both federal and non-federal hospitals) = 6.2 days (Table 108)[6]

  Total waste generated in year 2008 to 2009 =**1.34 million tonnes**

- World Health Organisation [111]
  Medical waste generation = 1.1 to 12.0 kg/capita
  Population of USA in 2010 = 304280000
  Hospital waste generation range = **0.3 million tonnes to 3.6 million tonnes**

- Hospital waste = 5.9 million tons = **5.35 million tonnes** [88]
- In 1994 , USA generated around 3.361 million tons of medical waste **= 3.05 million tonnes**[112]

  The higher estimate of **5.35 million tonnes** has been used in the study:
    - Due the futuristic perspective of nanomedicine waste
    - Increasing stringency in regulations concerning hospital waste

Table AF.T4.1 Data for aquatic toxicity. Data extracted from 12 related scientific papers. Ecological effects selected to create probabilistic species distribution were mortality and malformation, growth inhibition, reproductive impairment and acute immobilisation. Twenty three toxicity endpoints spread across four different taxonomic groups – fish, algae, crustacean and bacteria –were used to construct the Species Sensitivity Distribution for the aquatic compartment. The term Highest Observed No Effect Concentration (HONEC) was used when a range of concentrations were tested and the effects reported at the highest concentration tested was not statistically different from the control for the selected endpoint. The term No Observed Effect Concentration (NOEC) was used when two or less than two concentrations were tested and the reported concentration was not statistically significantly different from the control treatment. The concentration which caused an adverse effect in 10% of the test organisms was termed as Lowest Observed Effect Concentration (LOEC). The lowest concentration which caused an adverse effect in x% of the test organisms has been termed as EC_x or if x% of the test organisms died, it has been represented as LC_x. We used Assessment Factors (AF) to account for chronic toxicity (AF time) and to extrapolate to no observed effect values (AF-no effect) for deriving the species sensitivity values. For short time or acute exposure studies, the factor used for AF time was 10. AF no-effect factor used was 1 for the concentration which showed no difference in comparison to the control treatment, AF no-effect factor used was 2, if $L(E)C_{10} \leq L(E)Cx < L(E)C_{50}$ and a factor of 10 was used to derive NOEC if $L(E)_{50} \leq L(E)Cx \leq L(E)C_{100}$. Various units of exposure concentrations reported in the studies were standardised to microgram/litre.

Gold nanomaterial tested (particle size in nm and coating)	Test organism	Exposure concentrations	Toxic endpoint	Effect concentration (µg/L)	Type of toxicity test	AF time	AF no-effect	Species sensitivity values (µg/L)	Source
15-35 nm Capping: Poly vinyl alcohol	*Danio rerio*	10, 25, 50, 75, 100 µg/ml	Mortality	HONEC=100000	Acute toxicity test	10	1	10000	[113]
0.8 nmCapping -TMAT (N,N,N-trimethylammonium ethane thiol)	*Danio rerio*	(16, 80, 400 ppb), (2,10,50, 250) ppm	Mortality and malformation	EC_{60}=2000 ($p<0.01$)	Acute toxicity test (120 hpf)	10	10	20	[114]
1.5 nm Capping–TMAT (N,N,N-trimethyl ammonium ethane thiol)	*Danio rerio*	(16, 80, 400 ppb), (2,10,50, 250) ppm	Mortality and malformation	EC_{40} = 80 ($p<0.05$)	Acute toxicity test (120 hpf)	10	2	4	[114]
15 nm Capping -TMAT (N,N,N-trimethyl ammonium ethane thiol)	*Danio rerio*	(16, 80, 400 ppb), (2,10,50, 250) ppm	Mortality and malformation	EC_{40} = 50000 ($p<0.01$)	Acute toxicity test(120 hpf)	10	2	2500	[114]
0.8 nm Capping: 2-mercapto ethane sulfonic acid (MES)	*Danio rerio*	(16, 80, 400 ppb), (2,10,50, 250) ppm	Mortality and malformation	LOEC = 50000 ($p<0.01$)	Acute toxicity test(120 hpf)	10	2	2500	[114]
1.5 nm Capping: 2-mercapto ethane sulfonic acid (MES)	*Danio rerio*	(16, 80, 400 ppb), (2,10,50, 250) ppm	Mortality and malformation	LOEC = 2000 ($p<0.01$)	Acute toxicity test(120 hpf)	10	2	100	[114]
0.8 nm, 1.5nm, 15 nm capped with MEE (2,2 mercapto ethoxy ethanol) and MEEE (2-(2-(2-mercaptoethoxy)ethoxy) ethanol)	*Danio rerio*	(16, 80, 400 ppb), (2,10,50, 250) ppm	Mortality and malformation	HONEC =250000	Acute toxicity test(120 hpf)	10	1	25000	[114]
1.2 nm (3-mercaptopropionic acid-	*Danio rerio*	0.08 to 50 µg/ml	Mortality and	HONEC = 50000	Acute toxicity	10	1	5000	[115]

Gold nanomaterial tested (particle size in nm and coating)	Test organism	Exposure concentrations	Toxic endpoint	Effect concentration (µg/L)	Type of toxicity test	AF time	AF no-effect	Species sensitivity values (µg/L)	Source
functionalized)			malformation		test(120 hpf)				
3 nm (4-9 nm) Triphenylphosphine monosulfonate (TPPMS)-functionalised	*Danio rerio*	0.25,2.5,25,250 µM	Mortality, embryonic malformations	HONEC = 49000	Acute toxicity test (120 hpf)	10	1	4900	[116]
10 nm (14-21nm) TPPMS functionalised	*Danio rerio*	0.25,2.5,25,250 µM	Mortality, embryonic malformations	HONEC = 49000	Acute toxicity test(120 hpf)	10	1	4900	[116]
50 nm (31-60nm) TPPMS functionalised	*Danio rerio*	0.25,2.5,25,250 µM	Mortality, embryonic malformations	HONEC = 49000	Acute toxicity test(120 hpf)	10	1	4900	[116]
100 nm (75-115nm) TPPMS functionalised	*Danio rerio*	0.25,2.5,25,250 µM	Mortality, embryonic malformations	HONEC = 49000	Acute toxicity test(120 hpf)	10	1	4900	[116]
1.3 nm TMAT (N,N,N-trimethylammoniumethanethiol) functionalised	*Danio rerio*	0.08, 0.4, 2, 10, 20, 30, 40, and 50 mg/l	Mortality	LC50 = 30000	Acute toxicity test(120 hpf)	10	10	300	[117]
2 nm (alkane thiol-ethylene glycol and then functionalised A1- Hydrophilic positive charge	*Oryzias latipes (adult)*	20 nM of Au-NP (800-1000ppb of Au)	Mortality	NOEC = 973	Acute toxicity test (120 hrs)	10	1	97	[118]
2 nm (alkane thiol-ethylene glycol and then functionalised A2- Hydrophilic negative charge	*Oryzias latipes (adult)*	20 nM of Au-NP(800-1000ppb of Au)	Mortality	NOEC = 973	Acute toxicity test (120 hrs)	10	1	97	[118]
2 nm (alkane thiol-ethylene glycol and then functionalised ; A3- Hydrophilic neutral)	*Oryzias latipes (adult)*	20 nM of Au-NP(800-1000ppb of Au)	Mortality	NOEC = 973	Acute toxicity test (120 hrs)	10	1	97	[118]
2 nm (alkane thiol-ethylene glycol and then functionalised; A4- Hydrophobic positive charge)	*Oryzias latipes (adult)*	20 nM of Au-NP (800-1000ppb of Au)	Mortality	LC_{100} = 973	Acute toxicity test (24 hrs)	10	10	10	[118]
20 nm (15-21 nm) Capping: citrate	*Daphnia magna*	Not clear from the study	Acute immobilisation and reproductive test	LC_{50}=70000	Acute toxicity test	10	10	700	[119]

Gold nanomaterial tested (particle size in nm and coating)	Test organism	Exposure concentrations	Toxic endpoint	Effect concentration (µg/L)	Type of toxicity test	AF time	AF no-effect	Species sensitivity values (µg/L)	Source
4.6 nm Capping: Dodecanethiol coated with Amphiphilic Polymer (hydrophobic part -dodecylamine and a hydrophilic part, poly-sobutylene-alt-maleic anhydride).	*Pseudokirchneriella subcapitata*	0.0012 to 0.12 µM (0.46 to 46 mg/L)	Growth inhibition test	EC_{50} =7500	Acute toxicity test (24 and 48 hrs)	10	10	75	[120]
4.6 nm (4 to 5.5 nm) Capping: 10 kD PEG coating on the ampiphilic coating	*Pseudokirchneriella subcapitata*	0.0012 to 0.12 µM (0.46 to 46 mg/L)	Growth inhibition test	EC_{50} =39000	Acute toxicity test (24 and 48 hrs)	10	10	390	[120]
2 nm Capping: α-D-manno-pyranoside terminated PAMAM (polyamidoamine) dendrimer) G-0 generation	*Chlamydomonas reinhardtii*	6 and 12 ng/ml	Growth inhibition test	GI60 (48 hours) - 12 (p<0.01) or EC60 = 12	Acute toxicity test (24 and 48 hrs)	10	10	0.12	[121]
4 nm (5-9 nm) Capping: Citrate	*Caenorhabditis elegans*	0, 2.5, 5.5, 7, 15, and 30 mg/L	LC 10	LC_{10} = 5900	Acute toxicity test (24 hrs)	10	2	295	[122]
10 nm Capping: Citrate	*Photobacterium phosphoreum*	28 µg/ml	Decrease in bioluminescence	NOEC= 28000	Microtox test	10	1	2800	[123]
5.1 nm Capping: Bovine Serum Albumin	*Bacteria*	Not clear from the study	Toxicity test	EC_{50} = 2.68*10^6	Microtox test	10	10	26800	[124]

Table AF.T4.2 Data for terrestrial toxicity. Data extracted from one paper. Ecotoxicity endpoint data transformed to species sensitivity values as explained in Table SI.S4.1

Gold nanomaterial tested (particle size in nm and coating)	Test organism	Exposure concentrations	Toxic endpoint	Effect concentration	Type of test	AF time	AF no-effect	Species sensitivity value (µg/kg)	Source
20 nm (20.5±0.7 nm) Capping: Citrate	**Eisenia fetida (Adult and fully clitelate)**	5, 20, 50 mg Au/kg of dry mass soil	Reproductive performance	LOEC = 50 mg Au/kg	Long term test (56 days)	1	2	25	[125]
55 nm (54.9±0.7 nm) Capping: Citrate	**Eisenia fetida (Adult and fully clitelate)**	5, 20, 50 mg Au/kg of dry mass soil	Reproductive performance	LOEC = 20 mg Au/kg	Long term test (56 days)	1	2	10	[125]

S3:Alternate Scenarios

S3 details the scenario and possibilities:

1. Where modelled PEC of Au-NP is arrived at by considering 100% excretion of the therapeutic in waste water and is named as Scenario 2 (worst case)
2. Where the environment risk is estimated by comparing this worst case PEC with lethal endpoints and sub-lethal endpoints for the aquatic compartment
3. Where the environment risk is estimated by comparing the realistic scenario 1 (with accumulation of therapeutics in the body) and pSSDs with sublethal endpoint

Scenario 2 (worst case): PEC of Au-NP without accumulation of Au-NP (from drugs) in body, i.e., 100% excretion. Black values designate concentrations; grey values designate yearly increases in concentrations. Au-NP concentrations in surface water and sediments represent no and complete sedimentation respectively. The results are expressed up to three significant digits.

		UK				US				Units
		Mean	Mode	Q_{15}	Q_{85}	Mean	Mode	Q_{15}	Q_{85}	
**STP Effluent**		**980**	**930**	500	1,460	**310**	**300**	170	460	pg/L
**Surface water**		**1,040**	**600**	500	1,600	**11**	**8.1**	6.3	16	pg/L
**STP sludge**		**280**	**290**	230	320	**330**	**330**	300	370	µg/kg
**Sludge treated soil**		**650**	**670**	540	760	**280**	**280**	250	320	ng/kg·y
**Sediments**		**640**	**370**	300	990	**12**	**9.0**	7.0	17	ng/kg·y
**Hazardous waste**		**77**	**27**	24	130	**65**	**44**	20	110	µg/kg
**Medical WIP**	Fly ash	**260**	**30**	36	520	**260**	**33**	37	520	µg/kg
	Bottom ash	**200**	**24**	27	390	**200**	**24**	27	400	µg/kg
**Municipal WIP**	Fly ash	**30**	**29**	24	37	**90**	**87**	76	100	µg/kg
	Bottom ash	**23**	**22**	17	29	**68**	**64**	55	82	µg/kg

Scenario 1: PEC vs pSSD (with the PEC considering accumulation of Au-NP in body)

PEC vs pSSD for water with sublethal end points: The graphs don't overlap and hence could indicate no risk **from Au-NP**

Figure AF –F1 PEC vs pSSD in water with sublethal end points

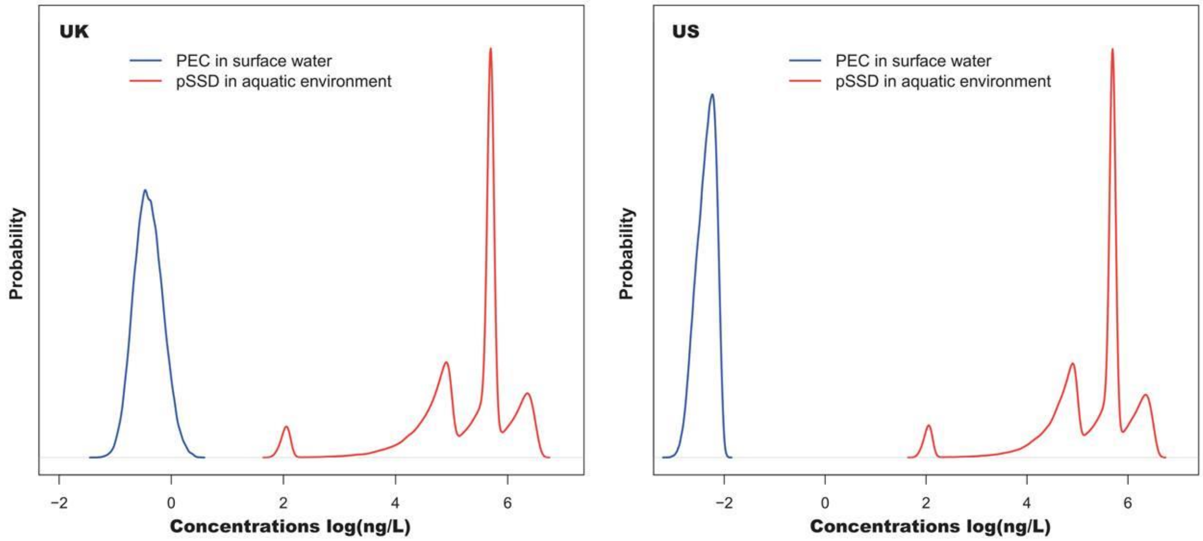

Scenario 2: PEC without accumulation of Au-NP in body and pSSD with lethal and sublethal endpoints

PEC vs pSSD for water and using lethal endpoints: The graphs don't overlap and hence could indicate no risk from Au-NP

Figure AF-F2 PEC vs pSSD for water and using lethal endpoints

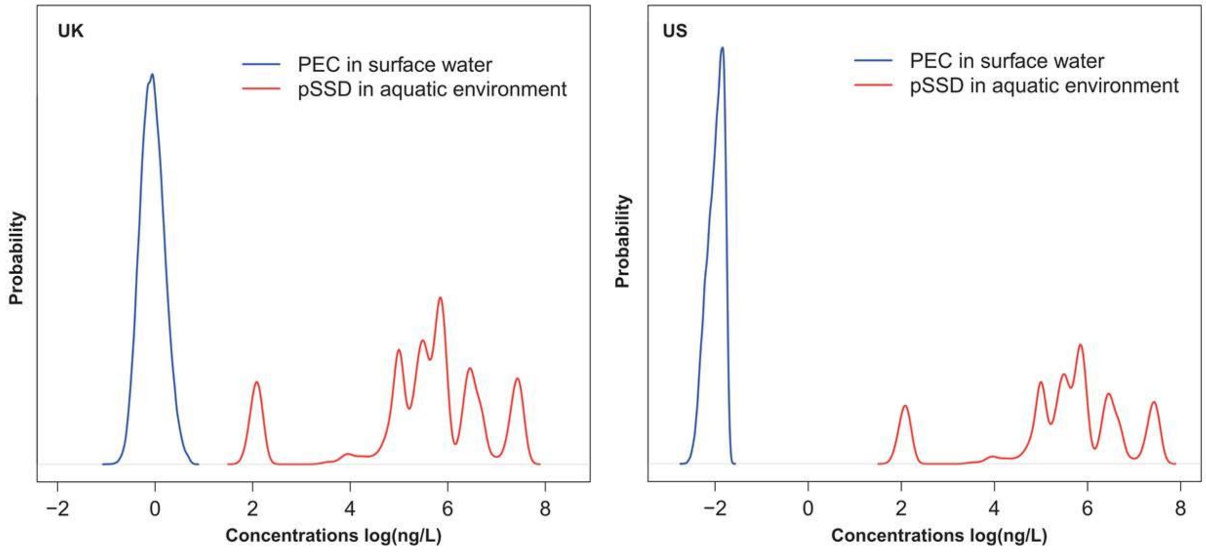

Figure AF-F3 PEC vs pSSD in soil with lethal endpoints: The graphs don't overlap and hence could indicate no risk from Au-NP

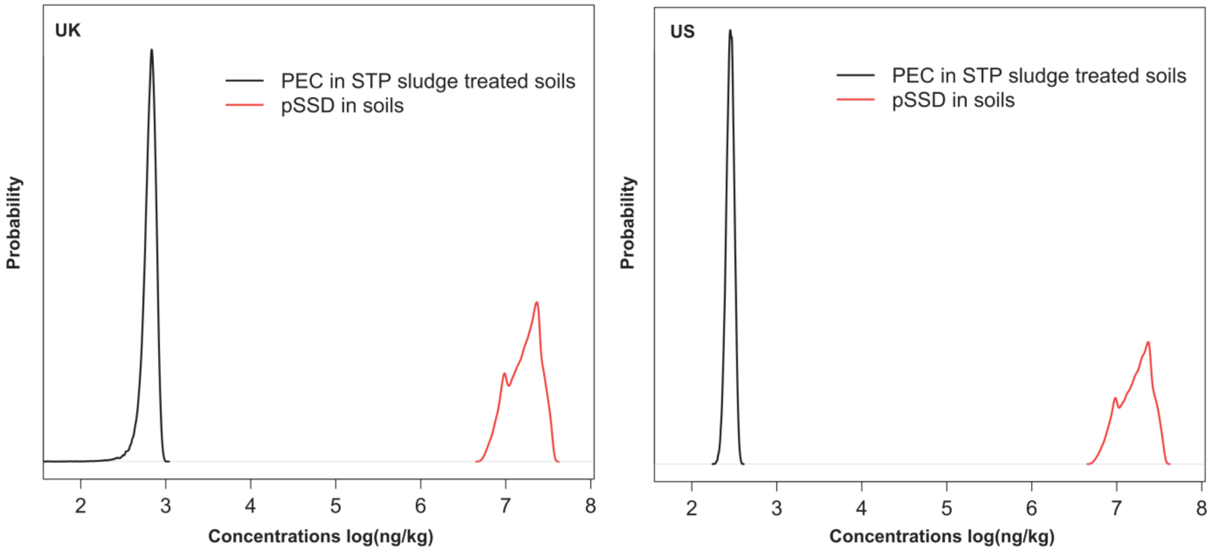

PEC vs pSSD for water using sublethal endpoints: The graphs don't overlap and hence could indicate no risk from Au-NP

Figure AF-F4 PEC vs pSSD in water with sublethal end points

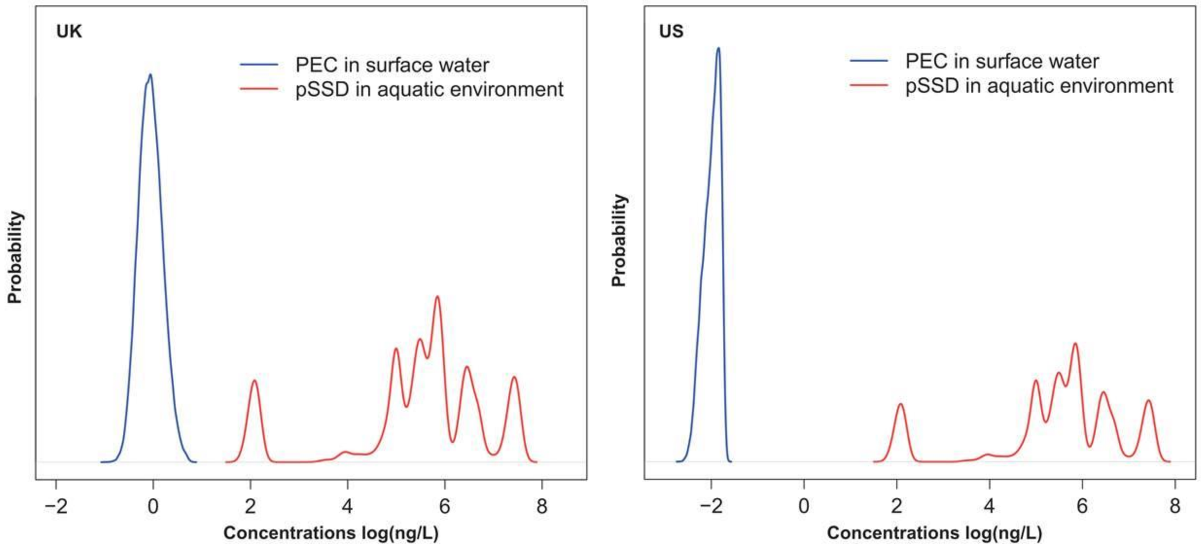

References

1. Nazareth A, Snowden T, Cheng YS, inventors; Church & Dwight Co., Inc., assignee. Diagnostic detection device patent US8211711 B2. 2012.

2. Wong RC, Tse HY, editors. Lateral Flow Immunoassay. Springer; 2009.

3. Zhou P, Lu Y, Zhu J, Hong J, Li B, Zhou J et al. Nanocolloidal gold-based immunoassay for the detection of the N-methylcarbamate pesticide carbofuran. J Agric Food Chem. 2004;52(14):4355-9.

4. BBI Solutions. Molar Concentration of Nanoparticles. http://www.bbisolutions.com/support/technical-information/molar-concentration-of-nanoparticles/. Accessed 19 December 2014.

5. BioAssay Works LLC. FAQ: Frequently Asked Questions. http://www.bioassayworks.com/faq.html. Accessed 07 January 2015.

6. National Centre for Health Statistics. Health, United States, 2011: With Special Feature on Socioeconomic Status and Health. Hyattsville, MD: US Department of Health and Human Services,2012. Report No.: DHHS Publication no. 2012-1232.

7. BIO-AMD. Why invest in BIAD? 2014. http://www.bioamd.com/investors. Accessed 13 May 2014.

8. U.S. Census Bureau. Age and Sex Composition: 2010 (2010 Census Briefs). online: U.S. Census Bureau2011. Report No.: C2010BR-03.

9. 2011 Census: Usual resident population by five-year age group and sex, United Kingdom [database on the Internet]. Office for National Statistics. 2012. Available from: http://www.ons.gov.uk/ons/publications/re-reference-tables.html?edition=tcm%3A77-270247. Accessed: 15 February 2013

10. Krutzik SR, inventor Trinity Biotech, assignee. Device and method for detecting polyvalent substances patent 6,653,066. Nov 25, 2003.

11. Rohrman BA, Leautaud V, Molyneux E, Richards-Kortum RR. A lateral flow assay for quantitative detection of amplified HIV-1 RNA. PloS one. 2012;7(9):21.

12. MedMira Laboratories Inc. Reveal™ Rapid HIV -1 Antibody Test (Package Insert). 2003. www.fda.gov/downloads/BiologicsBloodVaccines/BloodBloodProducts/ApprovedProducts/PremarketApprovalsPMAs/UCM091861.pdf. Accessed 21 May 2014.

13. Centers for Disease Control and Prevention. HIV testing. http://www.cdc.gov/hiv/topics/testing/index.htm. Accessed 30 May 2013 2013.

14. Yin Z, Brown AE, Hughes G, Nardone A, Gill ON, VC D et al. HIV in the United Kingdom: 2014 Report: data to end 2013. London: Public Health England2014 November 2014.

15. OraSure Technologies Inc. OraQuick® In-Home HIV Test. 2015. http://www.oraquick.com/. Accessed 31 December 2014.

16. Microphage Inc. KeyPath MRSA/MSSA Blood Culture Tes: Quick Reference Guide2013.

17. Elixhauser A, Friedman B, Stranges E. Statistical Brief 122: Septicemia in U.S. Hospitals, 2009. Agency for Healthcare Research and Quality, Rockville, MD. 2011. http://www.hcup-us.ahrq.gov/reports/statbriefs/sb122.jsp. Accessed 14 April 2014 2014.

18. PHE. Quarterly Epidemiological Commentary: Mandatory MRSA, MSSA and E. coli bacteraemia, and C. difficile infection data (up to October–December 2013). London: Public Health England2014.

19. Wilson M, Parkin I, Nair S, Gil-Tomas J, inventors; UCL Business PLC, assignee. Antimicrobial conjugates2008.

20. MRSAid. Nasal Decolonization of MRSA. http://www.mrsaid.com/for-clinicians/how-can-i-get-mrsaid/. Accessed 21 May 2014.

21. MRSAid. For clinicians: Treatment Protocol. http://www.mrsaid.com/for-clinicians/. Accessed 21 May 2014.

22. Gibson JD, Khanal BP, Zubarev ER. Paclitaxel-Functionalized Gold Nanoparticles. Journal of the American Chemical Society. 2007;129(37):11653-61. doi:10.1021/ja075181k.

23. von Eiff C, Becker K, Machka K, Stammer H, Peters G. Nasal Carriage as a Source of Staphylococcus aureus Bacteremia. New England Journal of Medicine. 2001;344(1):11-6. doi:doi:10.1056/NEJM200101043440102.

24. Deverick J. Anderson MM, Keith S. Kaye MD, David Classen MM, Kathleen M. Arias MC, Kelly Podgorny RMC, Helen Burstin MD et al. Strategies to Prevent Surgical Site Infections in Acute Care Hospitals • Infection Control and Hospital Epidemiology. 2008;29(S1):S51-S61. doi:10.1086/591064.

25. CDC. CDC/NCHS NATIONAL HOSPITAL DISCHARGE SURVEY. online. Maryland: Centers for Disease Control and Prevention2012 12 May 2014.

26. NHS Scotland. Table 2: Number of Procedures performed in acute hospitals (All Admission Types) Edinburgh: Information Services Division, NHS National Services Scotland2012 September 2012.

27. HSCIC. Hospital Episode Statistics for England. Inpatient statistics, 2011-12. Main procedures and interventions: summary. online: The Health and Social Care Information Centre2012.

28. NHS Wales. Principal Procedue Summary: PEDW Statistics - 2011/12: NHS Wales Informatics Sevice.

29. DHSSPS. Acute Programme of Care Total Operations Summary 2011/2012. online: Department of Health, Social Services and Public Safety2012.

30. Myers J, Stewart R, Smyth L. Northern Ireland Hospital Statistics: Inpatient And Daycase Activity Statistics 2011/12. online: Department of Health, Social Services and Public Safety2012 9 August 2012.

31. Ondine Biomedical Inc. User Manual: Photodisinfection System. online. Report No.: UG1000 Rev B.

32. Eke PI, Dye BA, Wei L, Thornton-Evans GO, Genco RJ. Prevalence of Periodontitis in Adults in the United States: 2009 and 2010. Journal of Dental Research. 2012;91(10):914-20. doi:10.1177/0022034512457373.

33. HSCIC. Adult Dental Health Survey 2009-Summary report and thematic series [NS]. online. U.K.: Health and Social Care Information Centre2011 March 24, 2011.

34. Morris AJ, Steele J, White DA. The oral cleanliness and periodontal health of UK adults in 1998. British dental journal. 2001;191(4):186-92. doi:10.1038/sj.bdj.4801135a.

35. NHS England. Improving dental care and oral health - A call to action evidence resource pack: NHS England Dental Analytical team2014 February 2014. Report No.: 01173.

36. Petersen PE, Ogawa H. Strengthening the prevention of periodontal disease: the WHO approach. Journal of periodontology. 2005;76(12):2187-93.

37. Raguse B, Chow E, Barton CS, Wieczorek L. Gold Nanoparticle Chemiresistor Sensors: Direct Sensing of Organics in Aqueous Electrolyte Solution. Analytical Chemistry. 2007;79(19):7333-9. doi:10.1021/ac070887i.

38. Steinecker WH, Sun Kyu K, Bohrer FI, Farina L, Kurdak C, Zellers ET. Electron-Beam Patterned Monolayer-Protected Gold Nanoparticle Interface Layers on a Chemiresistor Vapor Sensor Array. Sensors Journal, IEEE. 2011;11(2):469-80. doi:10.1109/JSEN.2010.2070063.

39. Haick H, Gang P, Adams O, inventors; Technion Research And Development Foundation Ltd., assignee. Detection of Cancer through Breath Comprising a Sensor Array Comprising Capped Conductive Nanoparticles2011.

40. Chow E, Müller K-H, Davies E, Raguse B, Wieczorek L, Cooper JS et al. Characterization of the Sensor Response of Gold Nanoparticle Chemiresistors. The Journal of Physical Chemistry C. 2010;114(41):17529-34. doi:10.1021/jp106055p.

41. Chow E, Herrmann J, Barton CS, Raguse B, Wieczorek L. Inkjet-printed gold nanoparticle chemiresistors: Influence of film morphology and ionic strength on the detection of organics dissolved in aqueous solution. Analytica Chimica Acta. 2009;632(1):135-42. doi:http://dx.doi.org/10.1016/j.aca.2008.10.070.

42. Cooper JS, Raguse B, Chow E, Hubble L, Muller KH, Wieczorek L. Gold nanoparticle chemiresistor sensor array that differentiates between hydrocarbon fuels dissolved in artificial seawater. Anal Chem. 2010;82(9):3788-95. doi:10.1021/ac1001788.

43. Raguse B, Barton CS, Müller K-H, Chow E, Wieczorek L. Gold Nanoparticle Chemiresistor Sensors in Aqueous Solution: Comparison of Hydrophobic and Hydrophilic Nanoparticle Films. The Journal of Physical Chemistry C. 2009;113(34):15390-7. doi:10.1021/jp9034453.

44. Lewis DJ, Day TM, MacPherson JV, Pikramenou Z. Luminescent nanobeads: attachment of surface reactive Eu(iii) complexes to gold nanoparticles. Chemical Communications. 2006(13):1433-5. doi:10.1039/B518091K.

45. Howlader N, Noone AM, Krapcho M, Garshell J, Miller D, Altekruse SF et al., editors. SEER Cancer Statistics Review, 1975-2011. Bethesda, MD: National Cancer Institute; 2014.

46. Centers for Disease Control and Prevention. Chronic Kidney Disease. http://www.cdc.gov/diabetes/programs/initiatives/kidney.html. Accessed 10 April 2014.

47. Nanosphere Inc. Verigene Technology. http://www.nanosphere.us/technology. Accessed 13 January 2015 2015.

48. Locado J, Gould C, Elixhauser A. Statistical Brief 124: Clostridium difficile Infections (CDI) in Hospital Stays, 2009. online. Rockville, MD: Agency for Healthcare Research and Quality2012.

49. Centers for Disease Control and Prevention. Seasonal Influenza Q&A. http://www.cdc.gov/flu/about/qa/disease.htm. Accessed 28 Dec 2014 2014.

50. Kujovich JL. Factor V Leiden thrombophilia. Genet Med. 2011;13(1):1-16.

51. ONS. Population Estimates by Ethnic Group 2002 – 2009: Office of the National Statistics2011 18 May 2011.

52. Centers for Disease Control and Prevention. Venous Thromboembolism in Adult Hospitalizations — United States, 2007–2009. 2012. http://www.cdc.gov/mmwr/preview/mmwrhtml/mm6122a1.htm. Accessed 23 May 2014.

53. NICE. Venous thromboembolism: reducing the risk of venous thromboembolism (deep vein thrombosis and pulmonary embolism) in patients admitted to hospital. London, U.K.: National Clinical Guideline Centre - Acute and Chronic Conditions 2009.

54. Hansen PW, Krauledat PB, inventors; Pointcare Technologies Inc, assignee. Enhanced cellular assay method for use in flow cytometry or similar instruments using optically resonant particles2004.

55. Hansen P, Barry D, Restell A, Sylvia D, Magnin O, Dombkowski D et al. Physics of a rapid CD4 lymphocyte count with colloidal gold. Cytometry Part A : the journal of the International Society for Analytical Cytology. 2012;81(3):222-31. doi:10.1002/cyto.a.21139.

56. CDC. HIV Surveillance Report, 2011. online. Atlanta, Georgia: Centers for Disease Control and Prevention,2013 February 2013. Report No.: Vol 23.

57. AVERT. Starting, Monitoring & Switching HIV Treatment. http://www.avert.org/starting-monitoring-switching-hiv-treatment.htm. Accessed 26 December 2014.

58. Paciotti GF, Myer L, Weinreich D, Goia D, Pavel N, McLaughlin RE et al. Colloidal Gold: A Novel Nanoparticle Vector for Tumor Directed Drug Delivery. Drug Delivery. 2004;11(3):169-83. doi:doi:10.1080/10717540490433895.

59. Libutti SK, Paciotti GF, Byrnes AA, Alexander HR, Gannon WE, Walker M et al. Phase I and Pharmacokinetic Studies of CYT-6091, a Novel PEGylated Colloidal Gold-rhTNF Nanomedicine. Clinical Cancer Research. 2010;16(24):6139-49. doi:10.1158/1078-0432.ccr-10-0978.

60. Paciotti G, Tamarkin L, inventors; Functionalized colloidal metal compositions and methods patent US2005175584. 11 August 2005.

61. Tsai D-H, Elzey S, DelRio FW, Keene AM, Tyner KM, Clogston JD et al. Tumor necrosis factor interaction with gold nanoparticles. Nanoscale. 2012;4(10):3208-17. doi:10.1039/C2NR30415E.

62. Cancer Reasearch UK. Bowel cancer statistics. http://www.cancerresearchuk.org/cancer-info/cancerstats/types/bowel/. Accessed 5 January 2015.

63. Cancer Reasearch UK. Pancreatic cancer statistics. http://www.cancerresearchuk.org/cancer-info/cancerstats/types/pancreas/. Accessed 5 January 2015.

64. Cancer Reasearch UK. Breast cancer statistics. http://www.cancerresearchuk.org/cancer-info/cancerstats/types/breast/. Accessed 5 January 2015.

65. Gad SC, Sharp KL, Montgomery C, Payne JD, Goodrich GP. Evaluation of the toxicity of intravenous delivery of auroshell particles (gold-silica nanoshells). International journal of toxicology. 2012;31(6):584-94. doi:10.1177/1091581812465969.

66. Cancer Reasearch UK. Lung cancer statistics. http://www.cancerresearchuk.org/cancer-info/cancerstats/types/lung/. Accessed 5 January 2015.

67. Cancer Research UK. Oral cancer statistics. http://www.cancerresearchuk.org/cancer-info/cancerstats/types/oral/. Accessed 5 January 2015.

68. Williams P, Rademacher T, Schobel AM, Dadey E, inventors; Combination peptide-nanoparticles and delivery systems incorporating same patent US2012/0301535. 29 Nov 2012.

69. Joshi SR, Parikh RM, Das A. Insulin-History, Biochemistry, Physiology and Pharmacology. JOURNAL-ASSOCIATION OF PHYSICIANS OF INDIA. 2007;55(L):19.

70. Centers for Disease Control and Prevention. National Diabetes Statistics Report: Estimates of Diabetes and Its Burden in the United States. online. Atlanta, GA: US Department of Health and Human Services; 20142014.

71. Diabetes UK. Diabetes: Facts and Stats. online. U.K.2014.

72. Water UK. Recycling of Biosolids to Agricultural Land. online: Water UK2010.

73. ECB. Technical Guidance Document on Risk Assessment: Part II. online. Luxembourg: European Chemicals Bureau, European Chemicals Bureau;2003. Report No.: EUR 20418 EN/2.

74. USDA. Major Uses of Land in the United States, 2007. USDA Economic Research Service. 2011. http://www.ers.usda.gov/data-products/major-land-uses.aspx#25984. Accessed 1 November 2013 2013.

75. Trading Economics. Arable land (hectares) in the United States. http://www.tradingeconomics.com/united-states/arable-land-hectares-wb-data.html. Accessed 23 March 2014.

76. UN-HABITAT. Global Atlas of Excreta, Wastewater Sludge, and Biosolids Management: Moving Forward the Sustainable and Welcome Uses of A Global Resource. online. Kenya: United Nations Human Settlements Programme2008.

77. OECD. Revised Emission Scenario Document for Wood Preservatives. Paris: Organisation for Economic Co-operation and Development (OECD), Publications OEHaS;2013 September 2013. Report No.: ENV/JM/MONO(2013)21.

78. ECHA. Guidance on information requirements and chemical safety assessment: Chapter R.16 Environmental exposure estimation. Helsinki, Finland: European Chemicals Agency2012 October 2012.

79. Morton RD, Rowland C, Wood C, Meek L, Marston C, Smith G et al. Final Report for LCM2007 - the new UK land cover map. online: NERC/Centre for Ecology & Hydrology (CEH Project Number: C03259)2011. Report No.: Countryside Survey Technical Report No 11/07.

80. U.S. Census Bureau. Statistical Abstract of the United States: 2012. online, Bureau USC;2013.

81. Gottschalk F, Sonderer T, Scholz RW, Nowack B. Modeled Environmental Concentrations of Engineered Nanomaterials (TiO2, ZnO, Ag, CNT, Fullerenes) for Different Regions. Environ Sci Technol. 2009;43(24):9216-22. doi:10.1021/es9015553.

82. Dedeh A, Ciutat A, Treguer-Delapierre M, Bourdineaud JP. Impact of gold nanoparticles on zebrafish exposed to a spiked sediment. Nanotoxicology. 2014;0(0):1-10. doi:10.3109/17435390.2014.889238.

83. DEFRA. Waste water treatment in the United Kingdom - 2012. online. London: Department for Enviroment, Food and Rural Affairs, Department for Enviroment FaRA;2012. Report No.: PB13811.

84. USEPA. Progress in Water Quality: An Evaluation of the National Investment in Municipal Wastewater Treatment. online. Washington DC: United States Environmental Protection Agecy2000. Report No.: EPA-832-R-00-008.

85. NEBRA. A National Biosolids Regulation, Quality, End Use and Disposal Survey: Final Report July 20, 2007. online. NH, USA: North East Biosolids and Residuals Association2007.

86. DEFRA. Environment Statistics - Key facts. online. London: Department for Environment, Food and Rural Affairs2013 January 2013. Report No.: PB 13671.

87. RTI International. Memorandum: Inventory of Hospital/Medical/Infectious Waste Incinerators Potentially Covered by the Proposed Section 111(d)/129 Federal Plan2012. Report No.: EPA Contract No. EP-D-07-019.

88. Practice Greenhealth. Waste. https://practicegreenhealth.org/topics/waste. Accessed 19 March 2014.

89. Kwakye G, Brat GA, Makary MA. Green surgical practices for health care. Archives of surgery. 2011;146(2):131-6. doi:10.1001/archsurg.2010.343.

90. Key Waste Streams: Municipal waste [database on the Internet]. European Commission. 2013. Available from:

http://epp.eurostat.ec.europa.eu/portal/page/portal/waste/key_waste_streams/municipal_waste. Accessed: 5 November 2013

91. Waste Generation and Management [database on the Internet]. European Commission. 2013. Available from: http://epp.eurostat.ec.europa.eu/portal/page/portal/waste/waste_generation_management. Accessed: 5 November 2013

92. USEPA. Municipal Solid Waste Generation, Recycling, and Disposal in the United States , Facts and Figures for 2011. online. Washington DC: United States Environmental Protection Agency2013. Report No.: EPA530-F-13-001.

93. USEPA. Clean Watersheds Needs Survey 2008: Report to Congress. online. Washington: United States Environment Protection Agency, Municipal Support Division SMB;2008. Report No.: EPA-832-R-10-002.

94. Ellis BJ, editor. Sewer Misconnections In England And Wales: Are They A Serious Problem? 7th International Conference on Sewer Processes & Networks; 2013 28-30 August 2013; Sheffield, U.K.

95. Accommodation type - Households, local authorities in the United Kingdom [database on the Internet]2011. Available from: http://www.statistics.gov.uk/hub/people-places/housing-and-households/households. Accessed: 14 March 2014

96. Ellis BJ, Revitt DM, Vollertsen J, Blackwood DJ. Factors influencing temporal exfiltration rates in sewer systems. 11th International Conference on Urban Drainage; 31 August - 5 September; Edinburgh, Scotland2008.

97. Rutsch M, Rieckermann J, Cullmann J, Ellis JB, Vollertsen J, Krebs P. Towards a better understanding of sewer exfiltration. Water Res. 2008;42(10–11):2385-94. doi:http://dx.doi.org/10.1016/j.watres.2008.01.019.

98. Ellis BJ, Revitt DM, Blackwood DJ, Gilmour D. Leaky sewers: Assessing the hydrology and impact of exfiltration in urban sewers. In: Webb B, Acreman M, Maksimovic C, Smithers H, Kirby C, editors. Hydrology: Science and Practice for the 21st Century Volume II. Krips, Netherlands: British Hydrological Society; 2004. p. 266-71.

99. Jr. Sharp JM. The impacts of urbanization on groundwater systems and recharge. 2010 doi:DOI 10.4409/Am-004-10-0008.

100. ASCE. 2013 Report Card for America's Infrastructure. online: American Society of Civil Engineers2013.

101. British Water. Code of Practice -Flows and Loads 4 - Sizing criteria, Treatment Capacity for Sewage Treatment Systems. online. London: British Water2013. Report No.: BW COP: 18.11/13.

102. Scott Wilson. Teignbridge Water Cycle Study: Outline Study. online. Devon: Teignbridge District Council2010.

103. Ellis BJ. Sewer infiltration/exfiltration and interactions with sewer flows and groundwater quality. 2nd International Conference on Interactions Between Sewers, Treatment Plants and Receiving Waters in Urban Areas; 19-22 February 2001; Portugal2001.

104. Rueedi J, Cronin AA, Morris BL. Estimation of sewer leakage to urban groundwater using depth-specific hydrochemistry. Water and Environment Journal. 2009;23(2):134-44. doi:10.1111/j.1747-6593.2008.00119.x.

105. National Biosolids Partnership. Potential Uses of Biosolids Fact Sheet2013.

106. Cremation Society of Great Britain. Progress of cremation in the United Kingdom 1885-2012. http://www.srgw.demon.co.uk/CremSoc4/Stats/National/ProgressF.html. Accessed 14 March 2014 2014.

107. National Funeral Directors Association. U.S. Cremation Statistics. National Funeral Directors Association. http://nfda.org/consumer-resources-cremation/78-us-cremation-statistics.html. Accessed 14 March 2014 2014.

108. Kagoma Y, Stall N, Rubinstein E, Naudie D. People, planet and profits: the case for greening operating rooms. CMAJ : Canadian Medical Association journal = journal de l'Association medicale canadienne. 2012;184(17):1905-11. doi:10.1503/cmaj.112139.

109. Brennan L, editor. The challenges of implementing sustainability programs. ASHES Annual Conference; 2009 20-24 September 2009; Reno, NV: American Society of Healthcare Environmental Services.

110. Rutala WA, Mayhall CG. Medical Waste. Infection Control and Hospital Epidemiology. 1992;13(1):38-48. doi:10.2307/30146966.

111. WHO. Safe management of wastes from healthcare activities. online. Geneva: World Health Organisation1999.

112. USEPA. United States Response UNEP Questionnaire for Paragraph 29 study Enclosure 4a April 2010. Revised May 2010. 2010. http://www.unep.org/chemicalsandwaste/Portals/9/Mercury/Documents/para29submissions/USA-Waste%20Incineration_revised%206-1-10.pdf. Accessed 19 March 2014.

113. Asharani PV, lianwu Y, Gong Z, Valiyaveettil S. Comparison of the toxicity of silver, gold and platinum nanoparticles in developing zebrafish embryos. Nanotoxicology. 2011;5(1):43-54. doi:doi:10.3109/17435390.2010.489207.

114. Harper SL, Carriere JL, Miller JM, Hutchison JE, Maddux BLS, Tanguay RL. Systematic Evaluation of Nanomaterial Toxicity: Utility of Standardized Materials and Rapid Assays. ACS Nano. 2011;5(6):4688-97. doi:10.1021/nn200546k.

115. Truong L, Zaikova T, Richman EK, Hutchison JE, Tanguay RL. Media ionic strength impacts embryonic responses to engineered nanoparticle exposure. Nanotoxicology. 2012;6(7):691-9. doi:10.3109/17435390.2011.604440.

116. Bar-Ilan O, Albrecht RM, Fako VE, Furgeson DY. Toxicity Assessments of Multisized Gold and Silver Nanoparticles in Zebrafish Embryos. Small. 2009;5(16):1897-910. doi:10.1002/smll.200801716.

117. Kim KT, Zaikova T, Hutchison JE, Tanguay RL. Gold nanoparticles disrupt zebrafish eye development and pigmentation. Toxicological sciences : an official journal of the Society of Toxicology. 2013;133(2):275-88. doi:10.1093/toxsci/kft081.

118. Zhu Z-J, Carboni R, Quercio MJ, Yan B, Miranda OR, Anderton DL et al. Surface Properties Dictate Uptake, Distribution, Excretion, and Toxicity of Nanoparticles in Fish. Small. 2010;6(20):2261-5. doi:10.1002/smll.201000989.

119. Li T, Albee B, Alemayehu M, Diaz R, Ingham L, Kamal S et al. Comparative toxicity study of Ag, Au, and Ag–Au bimetallic nanoparticles on Daphnia magna. Analytical and Bioanalytical Chemistry. 2010;398(2):689-700. doi:10.1007/s00216-010-3915-1.

120. Van Hoecke K, De Schamphelaere KA, Ali Z, Zhang F, Elsaesser A, Rivera-Gil P et al. Ecotoxicity and uptake of polymer coated gold nanoparticles. Nanotoxicology. 2013;7(1):37-47. doi:10.3109/17435390.2011.626566.

121. Perreault F, Bogdan N, Morin M, Claverie J, Popovic R. Interaction of gold nanoglycodendrimers with algal cells (Chlamydomonas reinhardtii) and their effect on physiological processes. Nanotoxicology. 2012;6(2):109-20. doi:doi:10.3109/17435390.2011.562325.

122. Tsyusko OV, Unrine JM, Spurgeon D, Blalock E, Starnes D, Tseng M et al. Toxicogenomic Responses of the Model Organism Caenorhabditis elegans to Gold Nanoparticles. Environ Sci Technol. 2012;46(7):4115-24. doi:10.1021/es2033108.

123. Barrena R, Casals E, Colón J, Font X, Sánchez A, Puntes V. Evaluation of the ecotoxicity of model nanoparticles. Chemosphere. 2009;75(7):850-7. doi:10.1016/j.chemosphere.2009.01.078.

124. Zheng H, Liu L, Lu Y, Long Y, Wang L, Ho KP et al. Rapid determination of nanotoxicity using luminous bacteria. Analytical sciences : the international journal of the Japan Society for Analytical Chemistry. 2010;26(1):125-8.

125. Unrine JM, Hunyadi SE, Tsyusko OV, Rao W, Shoults-Wilson WA, Bertsch PM. Evidence for Bioavailability of Au Nanoparticles from Soil and Biodistribution within Earthworms (Eisenia fetida). Environ Sci Technol. 2010;44(21):8308-13. doi:10.1021/es101885w.
